# A Proteomics Approach to Profiling the Temporal Translational Response to Stress and Growth

**DOI:** 10.1016/j.isci.2018.11.004

**Published:** 2018-11-05

**Authors:** Daniel A. Rothenberg, J. Matthew Taliaferro, Sabrina M. Huber, Thomas J. Begley, Peter C. Dedon, Forest M. White

**Affiliations:** 1 Department of Biological Engineering, Massachusetts Institute of Technology, Cambridge, MA 02139, USA; 2 The Koch Institute for Integrative Cancer Research, Massachusetts Institute of Technology, Cambridge, MA 02139, USA; 3 Department of Biology, Massachusetts Institute of Technology, Cambridge, MA 02139, USA; 4 Department of Biochemistry and Molecular Genetics, University of Colorado School of Medicine, Aurora, CO 80045, USA; 5 RNA Bioscience Initiative, University of Colorado School of Medicine, Aurora, CO 80045, USA; 6 College of Nanoscale Science and Engineering, State University of New York, Albany, NY 12203, USA; 7 Infectious Disease IRG, Singapore-MIT Alliance for Research and Technology, Singapore, Singapore; 8 Center for Precision Cancer Medicine, Massachusetts Institute of Technology, Cambridge, MA 02139, USA

**Keywords:** Functional Aspects of Cell Biology, Methodology in Biological Sciences, Proteomics

## Abstract

To quantify dynamic protein synthesis rates, we developed MITNCAT, a method combining multiplexed isobaric mass tagging with pulsed SILAC (pSILAC) and bio-orthogonal non-canonical amino acid tagging (BONCAT) to label newly synthesized proteins with azidohomoalanine (Aha), thus enabling high temporal resolution across multiple conditions in a single analysis. MITNCAT quantification of protein synthesis rates following induction of the unfolded protein response revealed global down-regulation of protein synthesis, with stronger down-regulation of glycolytic and protein synthesis machinery proteins, but up-regulation of several key chaperones. Waves of temporally distinct protein synthesis were observed in response to epidermal growth factor, with altered synthesis detectable in the first 15 min. Comparison of protein synthesis with mRNA sequencing and ribosome footprinting distinguished protein synthesis driven by increased transcription versus increased translational efficiency. Temporal delays between ribosome occupancy and protein synthesis were observed and found to correlate with altered codon usage in significantly delayed proteins.

## Highlights

• MITNCAT combines BONCAT, pSILAC, and TMT to quantify protein synthesis rates
• MITNCAT quantified up-regulation of protein folding chaperones during the UPR
• MITNCAT revealed EGF-driven protein synthesis in four distinct temporal waves
• MITNCAT identified delayed synthesis proteins with enriched rare codons

## Graphical Abstract

**Figure:**
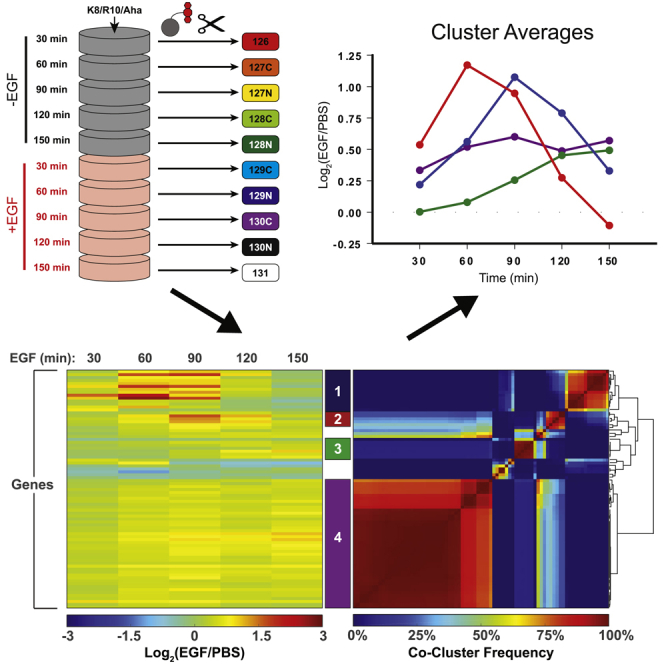

## Introduction

Cellular response to perturbation often leads to a change in cell state, accompanied by dynamic alterations in protein synthesis and degradation that ultimately result in changes in protein expression levels (Golan-Lavi et al., 2017). Measuring changes in mRNA abundance is commonly used to estimate changes in protein expression; however, relative mRNA abundance has been shown to be an incomplete predictor of protein synthesis and abundance (Schwanhäusser et al., 2011, Jovanovic et al., 2015) because translation is a highly regulated process that can be modulated by signaling pathways (Rowlands et al., 1988, Feng et al., 1992, Chen and London, 1995, Berlanga et al., 1999, Gingras et al., 2001, Novoa et al., 2003), RNA structural elements (Filbin and Kieft, 2009), and tRNA isoacceptor availability (Chan et al., 2010, Chan et al., 2012, Chionh et al., 2016).

Ribosome footprint (RFP) analysis, the identification of mRNA transcript fragments that are shielded by ribosomes, presumes that ribosome-bound transcripts are being translated into proteins and is considered the gold-standard RNA-based approach to estimate translation rates. RFP analysis involves the isolation and sequencing of ∼30 nucleotide mRNA fragments shielded by the ribosome from nuclease degradation (Ingolia et al., 2009, Ingolia, 2016). Since increased RFP abundance could result from increased ribosome density or increased transcript expression with constant ribosome density, normalizing RFP with transcript expression (typically measured by mRNA sequencing [mRNA-seq]) provides a metric known as translational efficiency (TE), effectively reading out the ribosome occupancy per transcript. Although RFP and TE provide a fairly accurate estimate of potential protein synthesis rates, these measurements do not account for stalled ribosomes and have been shown to be less representative of protein synthesis rates during cell stress response (Iwasaki and Ingolia, 2017, Liu et al., 2017).

Proteomics approaches quantify the protein product rather than the RNA precursors of protein synthesis. Two techniques, pulsed SILAC (pSILAC) (Schwanhäusser et al., 2009) and bio-orthogonal non-canonical amino acid tagging (BONCAT) (Dieterich et al., 2006), enable direct measurement of newly translated proteins. In pSILAC, heavy-isotope labeled amino acid analogs are added to cells in culture and are incorporated into newly synthesized proteins over a defined time period before mass spectrometry (MS)-based analysis. This approach allows for an estimation of protein turnover by comparing the abundance of the heavy, newly made peptides with the light, preexisting peptides (Doherty et al., 2005) and can be performed on up to two conditions simultaneously (Schwanhäusser et al., 2009). Owing to dynamic range and sensitivity limitations, it can be difficult to detect pSILAC-labeled, newly translated proteins against the large background of pre-existing proteins in the cell (Eichelbaum and Krijgsveld, 2014). These challenges effectively limit minimum incorporation time and make it difficult to monitor low-abundance proteins. However, targeted MS approaches such as multiple reaction monitoring have been used with pSILAC to quantify synthesis rates for selected proteins (Liu et al., 2017). In BONCAT, azidohomoalanine (Aha) (Dieterich et al., 2006), an azide-modified methionine analog used naturally by the native methionyl tRNA synthetase (MetRS) (Kiick et al., 2002), is added to cells and incorporated into newly synthesized proteins. The azide functional group on Aha enables selective enrichment through click chemistry-mediated solid-phase capture of Aha-labeled proteins. Combining BONCAT with pSILAC (e.g., BONLAC [Bowling et al., 2016]/QuanCAT [Howden et al., 2013] and HILAQ [Ma et al., 2017]) improves the sensitivity and coverage of pSILAC and provides a quantitative comparison of protein synthesis rates across two conditions (Eichelbaum et al., 2012). Using multiple MS analysis, this combined approach has been used for temporal analysis of newly synthesized proteins following macrophage activation (Eichelbaum and Krijgsveld, 2014). However, prolonged overlapping metabolic labeling periods prevented analysis of rapid changes in protein synthesis, and the use of pSILAC limited the number of time points assayed (Eichelbaum and Krijgsveld, 2014).

With the goal of developing a method that would allow for high sensitivity analysis of newly translated proteins at multiple time points with high temporal resolution, we developed MITNCAT (multiplex isobaric tagging/non-canonical amino acid tagging), combining BONCAT with pSILAC and using multiplexed isobaric tandem mass tagging (TMT) (Thompson et al., 2003) to quantitatively compare translation rates for thousands of proteins across ten different conditions in a single MS experiment. Here the combination of BONCAT and pSILAC enables enrichment for newly translated proteins and *post hoc* removal of non-specifically retained proteins from BONCAT enrichment, since newly translated proteins should all have pSILAC labels. Multiplex isobaric tagging generates quantification of newly synthesized proteins for discrete time bins within a single experiment. Previous studies have combined pSILAC and TMT to monitor protein turnover (Welle et al., 2016), whereas here we combine BONCAT, TMT, and pSILAC to describe the *temporal dynamics of protein synthesis rates at discrete time points following stimulation*. Application of MITNCAT to the unfolded protein response enabled the temporal analysis of thousands of protein synthesis rates and highlighted the differential translation regulation of a large number of metabolic and translational regulatory proteins.

We also applied MITNCAT to quantify protein synthesis rates following epidermal growth factor (EGF) stimulation of HeLa cells, a system whose dynamic response has been well characterized across miRNA (Avraham et al., 2010), transcript (Amit et al., 2007), protein expression (Waters et al., 2012, Shi et al., 2016, Golan-Lavi et al., 2017), and protein post-translational modification (Zhang et al., 2005, Reddy et al., 2016). Although the temporal dynamics at each of these levels has been shown to affect the cellular response to EGF, the effect on dynamic protein synthesis rates has yet to be characterized. Here we applied MITNCAT with discretely timed pulses of Aha and pSILAC to quantify the temporal dynamics of protein synthesis rates for thousands of proteins at multiple time points following EGF stimulation. These data document the temporal control of protein synthesis, including increased synthesis rates of dozens of proteins within the first 15 min, previously unprecedented temporal resolution. Comparison of proteomic synthesis rate data to mRNA-seq and RFP data at each time point established the transcriptional versus translational efficiency-based control of protein synthesis. Furthermore, we compared the temporal dynamics of protein synthesis and RFP, uncovering a potential role of codon bias in regulating temporal delays in protein synthesis.

## Results

### Isobaric Mass Tags Allow for Robust Multiplexed Quantitative Analysis of Newly Synthesized Proteins

Several publications over the past decade have documented the dynamic regulation of miRNA, mRNA, and protein expression following cell stimulation. Realizing that protein synthesis rates are likely similarly dynamic, we developed MITNCAT, a method to accurately quantify the temporal dynamics of protein synthesis rates at a global scale. In MITNCAT, multiplex isobaric tags enable the simultaneous analysis of newly synthesized proteins from multiple discrete time points following cellular stimulation. Briefly, Aha-based BONCAT is used to label newly synthesized proteins with a bio-orthogonal chemical handle and heavy-isotope labeled arginine and lysine pSILAC amino acids are added to the media concurrently with Aha. Proteins synthesized during the labeling period therefore incorporate Aha (for enrichment) and pSILAC (as a marker for specificity). Aha-labeled proteins are solid-phase captured onto a dibenzocyclooctyne (DBCO)-functionalized resin through a copper-free click chemistry reaction. After multiple rounds of highly stringent washes, the bound proteins are digested, on bead, to liberate pSILAC-labeled peptides. In the same tube, multiplex isobaric tandem mass tags are added to each sample to allow for quantitative MS analysis. The samples are then combined, subjected to high pH reverse phase fractionation, and analyzed via liquid chromatography-tandem mass spectrometry (LC)-MS/MS for discovery-mode, quantitative measurement of temporal dynamics of protein synthesis rates.

**Figure 1:**
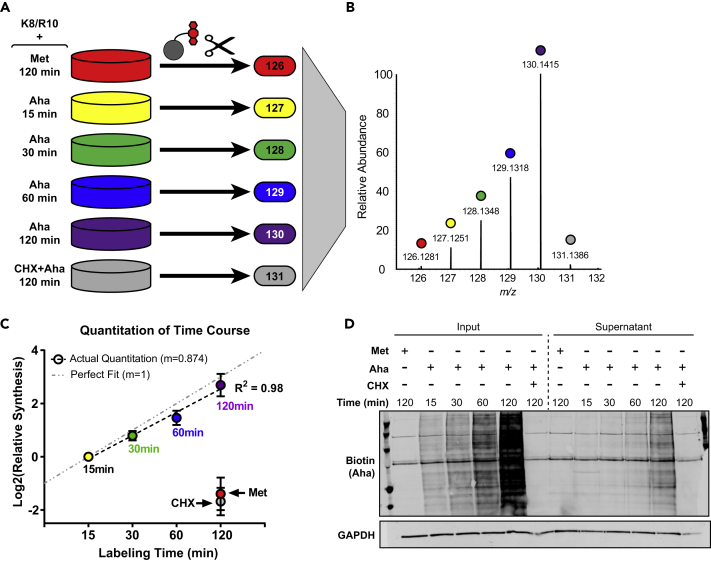
Analysis of Aha Labeling Time Course Demonstrates Reliable Quantitation of Newly Synthesized Proteins across Large Dynamic Range Newly synthesized proteins were labeled with Aha and pSILAC for variable amounts of time, enriched onto a DBCO-functionalized resin, digested with trypsin, and eluted peptides were labeled with isobaric mass tags (A); TMT reporter ions used for quantitation from exemplar spectra (B); a fitted line to the medians of all Aha-labeled samples demonstrates accuracy of quantification over a broad dynamic range (C); western blot for biotinylated Aha residues of newly synthesized proteins in inputs before click enrichment (left) and in the supernatant following click enrichment (right) (D). Data are median ± SEM.

To validate the accuracy of this quantitative approach to measure newly translated proteins, we chose a model system, MCF10a cells, and incubated the cells with 3 mM Aha for increasing periods of time: 15, 30, 60, and 120 min. Assuming that proteins were being synthesized at a continuous rate over this experiment, Aha-labeled newly synthesized proteins should increase linearly with time. Two negative controls were included in this experiment: methionine (Met) was substituted for Aha to assess the level of background binding, and 300 μg/mL cycloheximide (CHX) was added to the media 30 min before labeling with Aha for 120 min (Figure 1A, left) to determine if blocking translation could prevent Aha incorporation into proteins. Peptides were labeled with one of six different tandem mass tags (Figure 1A, right) that allow for relative quantitation of newly translated proteins among the different samples. The samples were then combined, fractionated (see Methods), and analyzed via LC-MS/MS. During fragmentation in MS/MS scans, low mass reporter ions are liberated for quantitation (Figure 1B). Peptides were identified to be from a newly synthesized protein only if they contained either a SILAC-labeled residue or an Aha residue (proteins could be labeled with more than one Aha residue, and not all Aha residues were necessarily subjected to the click reaction because of steric hindrance).

Since TMT-based quantification is relative and not absolute, the 15-min Aha-stimulated channel was used for normalization. Taking the median of all proteins observed in each channel, a more than 2-fold decrease was observed in the Met and CHX samples compared with the 15-min Aha-labeled sample, whereas the 30-, 60-, and 120-min labeled Aha channels each featured an increase in intensity that corresponded with the increase in duration of Aha labeling (Figure 1C). A line fitted to the four Aha-labeled time points has a slope of 0.87, slightly below the expected value of 1. This slight compression in the MS-based quantification was repeated in a subsequent replicate (Figure S1A). To determine the source of this deviation, we used an orthogonal, fluorescence-based method to quantify Aha-labeled proteins from each time point. An aliquot of the input and supernatant from each pull-down was analyzed by reacting each protein sample with DBCO-biotin to click on a biotin tag. Quantitative western blotting was then performed using a fluorophore-conjugated streptavidin as a probe. This analysis revealed time-dependent increases in Aha-labeling with a slope of 1 (Figure S1B) and robust depletion of Aha-labeled proteins from the supernatant (Figure 1D), suggesting that the decreased slope of the MS-based quantification is mostly likely not due to translation suppression associated with the decreased incorporation of Aha in place of Met (Kiick et al., 2002) but may instead be due to suppression of dynamic range in MS-based quantification (Savitski et al., 2013). Individual proteins, although showing increased variation, still demonstrate a robust linear increase in protein synthesis in response to longer labeling periods (Figure S1C). Stratifying proteins based on total reporter ion intensity reveals that proteins with the top 10% most abundant report ions have a slope of nearly 1, whereas the bottom 10% has a slope of 0.77 (Figure S1D), further suggesting compression due to isolation interference. Increasing the degree of sample fractionation could potentially improve the dynamic range, but given that we were able to fairly accurately quantify an 8-fold change in translation rate, we determined that the current approach should be suitable for further applications. Stratifying proteins based on degradation kinetics (McShane et al., 2016) yielded no differences (Figure S1E), suggesting that protein degradation kinetics do not play a major role in quantification on the timescales tested.

### Analysis of Protein Synthesis during the Unfolded Protein Response Shows an Up-regulation of Stress Proteins in the Background of Global Translational Repression

To validate the robustness of this method in a biological context, we selected the unfolded protein response (UPR), a stress response known to drive inhibition of protein synthesis to prevent further protein misfolding while also up-regulating protein folding chaperones to aid in the repair of misfolded proteins.

To induce the UPR, 10 μg/mL tunicamycin was added to cells and newly synthesized proteins were labeled with Aha, K8, and R10 for 30 min starting at either 0, 1, 2, 3 or 4 hr after treatment (Figure S2A). DMSO-treated controls were collected at matched time points to account for differences in starvation length as well as any artifacts that may arise owing to the presence of Aha (Figure S2A).

**Figure 2:**
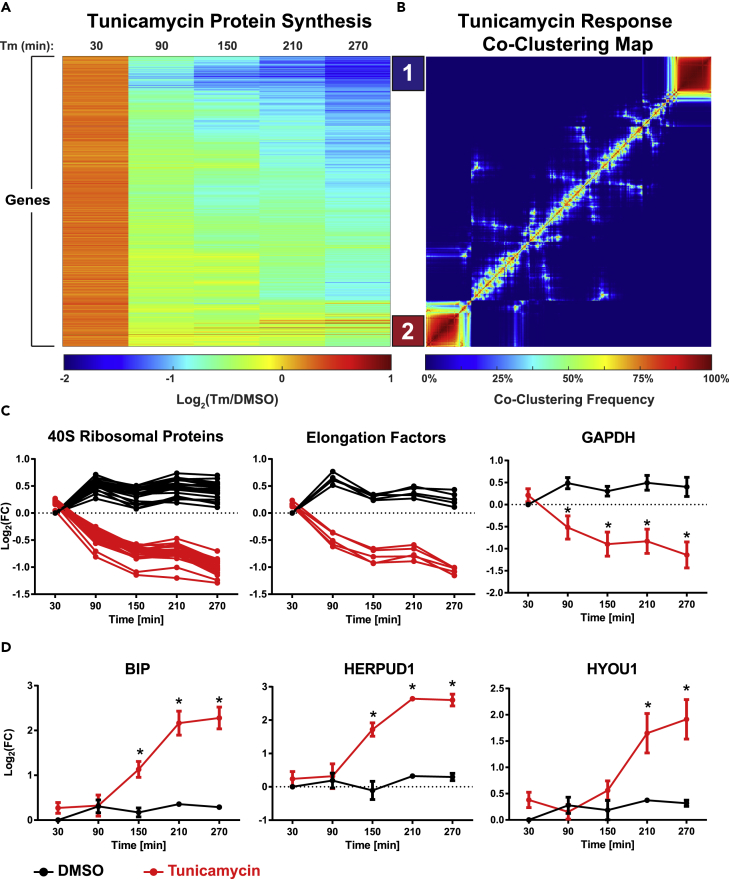
Temporal Profiles of Protein Synthesis during the Unfolded Protein Response following Tunicamycin Treatment Temporal changes in protein synthesis over the first 5 hr following induction of the UPR by tunicamycin (A) were clustered using self-organizing maps, revealing two distinct groups (B); cluster 1 is down-regulated below the median and contains proteins associated with translational machinery and glycolysis (C); cluster 2 is up-regulated above the median and contains proteins associated with an acute stress response (D). n = 3 biological replicates. Data are mean ± SEM, * = p < 0.05.

MITNCAT led to the quantification of a total of 3,178 unique proteins, with 2,007 unique proteins appearing in at least two of the three replicates and therefore considered for subsequent analyses. When comparing the median of log2-transformed time points, we observed a global decrease in protein synthesis starting between 30 and 90 min following tunicamycin treatment compared with DMSO controls (Figures 2A and S2B). This observation is consistent with previous reports showing that global translation decreases owing to phosphorylation of eIF2α at approximately 30 min following endoplasmic reticulum (ER) stress (Novoa et al., 2003).

All proteins were subjected to clustering using a self-organizing map. To identify the most robust clusters, this process was repeated 1,000 times using random initial seeds to generate a co-clustering frequency map (Figure 2B). This approach led to the identification of two distinct clusters: one characterized by protein synthesis down-regulated below the median (cluster 1, Figure S2C) and one characterized by protein synthesis up-regulated above the median (cluster 2, Figure S2C).

Gene ontology (GO) term analysis revealed that the down-regulated cluster 1 is statistically significantly enriched for the terms *protein targeting to the ER* (p = 2.87 × 10^−20^), *translational initiation* (p = 2.80 × 10^−19^), and *ribosome biogenesis* (p = 7.15 × 10^−10^). The down-regulation of proteins involved in translation, such as ribosomal proteins, translation initiation factors, and translation elongation factors, (Figure 2C, left and center) may enhance the suppression of protein synthesis in cells undergoing the UPR.

The down-regulated cluster also contained proteins associated with glycolysis. The down-regulation of proteins involved in glycolysis, including GAPDH (Figure 2C, right), ENO1, TPI1, PKM, and ALDOA (Figure S2D), suggests that metabolic flux through the glycolytic pathway may be reduced during the UPR. It has been previously reported that tunicamycin treatment reduces glucose uptake, lactate production, and ATP levels (Wang et al., 2011), consistent with the observed down-regulation of proteins associated with these pathways. Interestingly, proteins involved in the tricarboxylic acid cycle, such as citrate synthase, SDHA, and IDH3, were not included in this down-regulated cluster (data not shown).

Cluster 2 contained proteins that were down-regulated to a lesser extent and also included proteins that were up-regulated in the background of global translation repression. One of the most highly up-regulated proteins in this cluster was BIP, which is the canonical ER stress response chaperone. Other proteins that exhibited a large increase in translation rate following tunicamycin treatment were HERPUD1, which is involved in targeting proteins for degradation via the ERAD pathway, and HYOU1, another member of the heat shock family of proteins involved in protein folding and cell survival in response to stress (Figure 2D). Other stress-related proteins were also observed to be up-regulated (Figure S2E).

Since the inhibition of translation during the UPR is mediated by the phosphorylation of eIF2α by PERK, we hypothesized that the observed overall repression of translation would not be seen by measuring RNA transcript abundance. Indeed, mRNA-Seq did not recapitulate this global down-regulation of protein synthesis measured by MITNCAT (Figures S2F–S2H), as only selected transcripts were affected by tunicamycin treatment. Altogether, these data demonstrate the reliability of MITNCAT for quantifying the time course of protein synthesis changes at a global level in a complex biological system.

### EGF Stimulation Results in Temporally Distinct Waves of Protein Synthesis

It has been shown that epidermal growth factor receptor (EGFR) activation leads to temporally distinct waves of transcription, in which immediate-early genes (IEGs) are followed by delayed early genes (DEGs) and finally late response genes (LRGs), which are up-regulated 2 to 4 hr after stimulation (Amit et al., 2007, Avraham and Yarden, 2011, Feldman and Yarden, 2014). To determine whether temporally distinct waves also occurred for protein synthesis, we applied MITNCAT to quantify proteome-wide protein synthesis rates temporally distinct time windows following EGF stimulation. HeLa cells were serum starved for 24 hr and stimulated with 20 nM EGF, and Aha, K8, and R10 were concurrently applied in consecutive 30-min windows following EGF addition, resulting in time points collected at 30, 60, 90, 120, and 150 min (Figure S3A). To account for the effects of the KRM-free media starvation and Aha labeling, matched PBS controls were also collected at the same time points.

**Figure 3:**
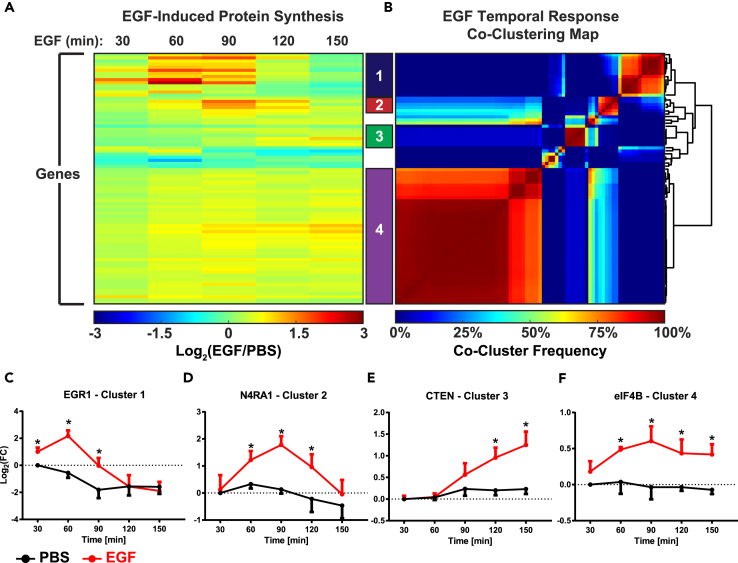
Changes in Protein Synthesis following EGF Stimulation Were Clustered into Four Groups Based on Temporal Behavior Temporal changes in protein synthesis over the first 150 min following EGF stimulation (A) were clustered by k-means clustering, resulting in four groups that have distinct temporal profiles (B). Exemplar proteins demonstrate the unique protein synthesis profiles of each group (C–F). n = 4 biological replicates. Data are mean ± SEM, * = p < 0.05.

Following processing and analysis, 1,749 unique proteins were identified across four replicates, with 1,007 unique proteins observed in at least two replicates and retained for subsequent analysis. To visualize the temporal dynamics of protein synthesis following EGF stimulation, proteins whose synthesis was statistically significantly altered in at least one time point were subjected to hierarchical clustering. The resulting heatmap highlights the temporal dynamics of synthesis of selected proteins, with some proteins peaking at 60–90 min post-treatment, whereas others feature increased synthesis as early as within the first 30 min of EGF stimulation (Figure 3A). Although there was a trend toward increased global protein synthesis following EGF stimulation, this trend did not reach statistical significance (Figure S3B).

To group proteins into temporally distinct clusters, k-means clustering analysis was performed using six clusters and Pearson correlation as the distance metric. To identify the most robust clusters, this process was repeated 10,000 times using a random initial seed; the results of this analysis were then plotted on a co-clustering frequency map (Figure 3B). This analysis revealed four distinct clusters, each with a unique temporal profile (Figure S3C).

Cluster 1 was characterized by increased protein synthesis as early as 30 min following EGF stimulation, with maximum synthesis at 60 min before returning to baseline levels by 150 min. This cluster contained many of the canonical IEGs, including EGR1 (Figure 3C), JUN, CYR61, and IER2 (Figure S3D), consistent with a model of rapid up-regulation of these genes within the first hour following stimulus. Intriguingly, this cluster also contained many of the canonical DEGs, such as DUSP1, ATF3, and ZFP36L1 (Figure S3D). This rapid up-regulation of DEGs was in contrast to the established literature that shows most DEG transcripts being up-regulated between 1 and 2 hr following EGF stimulation (Avraham and Yarden, 2011, Feldman and Yarden, 2014). One notable IEG absent from the dataset was MYC, which has previously been shown to be up-regulated on the transcript level in response to EGF stimulation (Amit et al., 2007). MYC was modestly up-regulated between 30 and 90 min, but not to an extent that was statistically significant in this analysis. This observation suggests that previously observed increases in MYC protein levels could be due to a combination of increased stability (Sears et al., 2000) and a modest increase in protein synthesis.

In response to EGF stimulation, synthesis of proteins in cluster 2 increased at 60 min, was maximal at 90 min, and subsequently decreased back toward basal levels. Similar to cluster 1, cluster 2 contained IEGs such as NR4A1 (Figure 3D) and NR4A3 and the DEG KLF10 (Figure S3E). Within clusters 1 and 2, synthesis of both IEGs and DEGs were observed between 30 and 90 min following EGF stimulation. Rather than showing a separate wave of IEGs followed by a wave of DEGs, as has been implicated by transcriptional analysis, IEGs and DEGs appeared to be collectively expressed within the same time frame with maximal synthesis occurring between 60 and 90 min following EGFR activation.

Cluster 3 was characterized by a delayed response to EGFR activation, with protein synthesis beginning at around 90 min and increasing through the final time point, 150 min after stimulation. This cluster contained several LRGs involved in cytoskeletal dynamics and cell motility, such as CTEN (Figure 3E), VASP, EZR, and EPPK1 (Figure S3F).

Finally, cluster 4 was characterized by an increase in synthesis beginning around 30 min and remaining elevated across all time points. This cluster almost exclusively contained proteins associated with the translational machinery, including ribosomal proteins, translation initiation factors such as eIF4B (Figure 3F), and translation elongation factors (Figure S3G). These data suggest that, in response to pro-growth cues such as EGF stimulation, cells increase their translational capacity by synthesizing more ribosomal proteins and associated translation factors to further increase synthesis of new proteins. This observed increase in ribosomal protein synthesis is consistent with the observation that ribosomal RNAs (rRNAs) also show an increase in transcription within 30 min following EGFR activation (Stefanovsky et al., 2001).

### Increasing Sampling Frequency Yields New Insights on Protein Synthesis Dynamics

**Figure 4:**
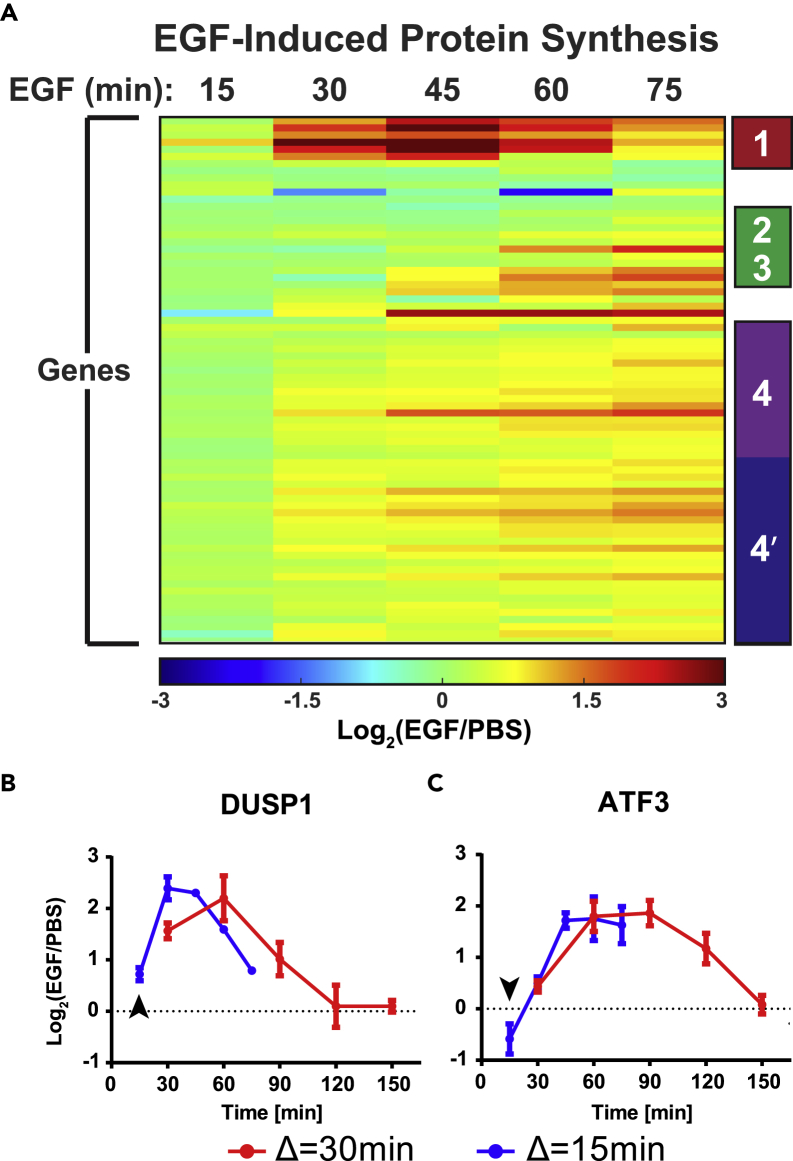
Decreasing the Duration of Aha Labeling Increased Temporal Resolution and Yielded New Insights into Protein Synthesis Dynamics Temporal changes in protein synthesis were sampled every 15 min over the first 75 min and clustered by k-means clustering. Clusters 2 and 3 from Figure 3 are now grouped into a single cluster, and cluster 4 bifurcates into two clusters based on changes within the first 15 min (A). Increasing temporal resolution allows for insights on protein changes within the first 15 min following EGF stimulation (B and C, arrowheads). n = 3 biological replicates. Data are mean ± SEM.

To better characterize the temporal response to EGF, especially for proteins that displayed dynamic protein synthesis such as those in clusters 1 and 2, we tested whether we could increase the temporal resolution of MITNCAT. Aha/K8/R10 labeling times were reduced from 30 min to 15 min, with samples collected at 15, 30, 45, 60, and 75 min following EGF treatment along with matched negative controls as described previously. In this analysis, 1,857 proteins were identified in total, with 1,135 proteins identified in two or more replicates. Somewhat to our surprise, decreasing labeling time to 15 min did not lead to a significant decrease in the number of identified proteins relative to the 30-min labeling experiments, suggesting that the dynamic range of the experiment (the difference between the most abundant and least abundant detectable proteins), rather than the overall sensitivity, might be the limiting factor in number of identified proteins. To visualize the dynamic profiles of protein synthesis, proteins with significantly altered synthesis following EGF stimulation were clustered to generate a heatmap (Figure 4A). This heatmap demonstrates that some proteins are rapidly synthesized within 15 min of EGF stimulation, whereas many additional proteins feature a strong increase between 30 and 60 min post-treatment. Analysis of this 15-min temporal resolution data by k-means clustering yielded similar clusters as compared with the 30-min resolution data. However, because the time course ended at 75 min, before cluster 2 proteins and LRGs in cluster 3 reach their maximum, LRGs and late IEG/DEGs previously found in clusters 2 and 3 were clustered together. Additionally, although ribosomal proteins and translation factors from cluster 4 remained clustered, the improved temporal resolution of the analysis enabled a bifurcation (clusters 4 and 4*) between those proteins that demonstrated an increased synthesis by 15 min compared with those that increased synthesis starting at 30 min (Figure S4A).

Despite the relatively small changes in overall temporal clusters, increasing temporal resolution provided interesting insights at specific time points. For instance, 24 proteins, including DUSP1 (Figure 4B, arrowhead), JUN, and several ribosomal proteins, demonstrated significantly increased protein synthesis as early as 15 min following EGF stimulation. Of these proteins, DUSP1 was one of the fastest responders, with expression increasing nearly 60% within the first 15 min. This rapid up-regulation of a DEG, even before most IEGs, has not been previously observed and stands in contrast to the classical model of DEGs being up-regulated following the expression of IEGs. The ATF3 transcription factor was also notable, as improved temporal resolution highlighted an immediate-early decrease below basal levels in ATF3 synthesis rates at 15 min following EGF stimulation, followed by an increase in synthesis at 30 min that peaked between 45 and 60 min (Figure 4C, arrowhead). This immediate down-regulation of ATF3 is corroborated by RFP analysis (Figure S5B, Table S5) and has not been previously observed.

### Comparison of Protein Synthesis to RNA Abundance and Ribosome Footprints Reveal Transcriptionally and Translationally Controlled Groups of Proteins

Increased synthesis of a given protein could be due to many different factors, including an increase in the abundance of the protein-coding transcript or an increase in translation efficiency due to increased number of ribosomes binding to each transcript or the rate at which the ribosomes move along the transcript. To determine the relationship between dynamic transcript expression, ribosome binding, and protein synthesis rates, we performed mRNA-seq and RFP on identical samples corresponding to the 30-, 60-, and 90-min time points following EGF stimulation. TE rates were calculated based on the mRNA-seq and RFP data and represent ribosome occupancy normalized to transcript abundance (see Methods). These data were then compared with the quantitative protein synthesis data at these same time points.

**Figure 5:**
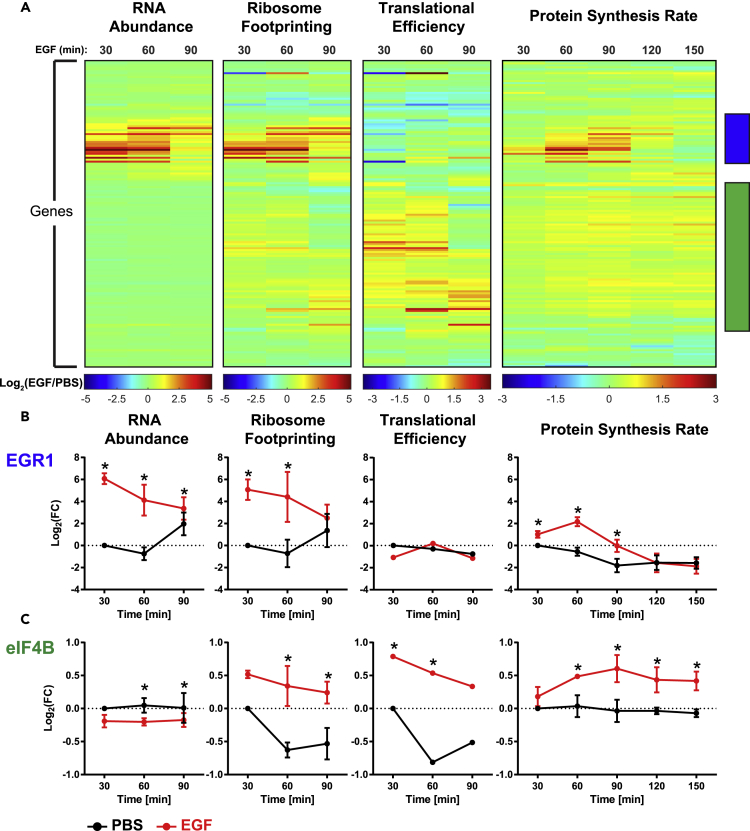
Temporal Response to EGF Stimulation Compared between Transcript Expression, Ribosome Binding, and Protein Synthesis Network-wide temporal response to EGF stimulation was assessed at the transcript expression level by mRNA-seq (n = 2), whereas translation rates were estimated by RFP (n = 2) and protein synthesis rates were measured by the MITNCAT approach (n = 3) (A). Comparing these datasets revealed a set of proteins whose altered synthesis correlated with changes in RNA abundance (blue bar), exemplified by EGR1 (B), suggesting regulation at transcription. Another group demonstrates changes in synthesis rates correlating with changes in translational efficiency (green bar), exemplified by eIF4B (C), suggesting regulation at translation. Data are mean ± SEM.

On comparison of protein synthesis with RNA abundance and TE measurements, two distinct groups of genes emerged (Figures 5A and S5A). In the first group, changes in protein synthesis correlated strongly with changes in RNA abundance (Figure 5A blue bar and Figure S5B), suggesting regulation predominantly at the level of transcription. This group contains most of the IEGs and DEGs that are transiently expressed at high levels, such as EGR1 (Figure 5B). This rapid up-regulation of EGR1 was also orthogonally confirmed by western blot, with new EGR1 protein increasing 1.9-fold from 30 to 60 min after EGF treatment (Figure S5C), compared with a 2.2-fold increase as measured by MITNCAT over that time (Figure 5B). Furthermore, co-treatment with proteasome inhibitor MG132 yields no change in newly synthesized EGR1 (Figure S5C), demonstrating that the difference in magnitude between mRNA/RFP levels and protein synthesis is not due to EGR1 protein degradation. In the second group, changes in protein synthesis correlated with variations in TE, with minimal change in RNA abundance (Figure 5A, green bar and Figure S5D), suggesting regulation on the level of translation. This cluster was highly enriched for ribosomal proteins and several translation initiation and elongation factors such as eIF4B (Figure 5C). These proteins were rapidly up-regulated and sustained throughout the duration of the analysis, albeit to a lesser magnitude than the IEGs, DEGs, and LRGs.

One notable difference between the two clusters was the magnitude of the change in protein synthesis. In the transcriptionally driven cluster, protein synthesis increased as high as 7-fold, whereas the most highly up-regulated protein in the translationally driven cluster experienced a 2-fold change in synthesis. Up-regulation solely through increased translation may be limited by the maximum rate at which ribosomes can bind and translate a transcript. Larger changes in protein expression may require increasing the number of mRNA transcripts available to be translated. This observation highlights a potential trade-off between fast but limited up-regulation through translational control and slower but more potent up-regulation through transcriptional control.

### Codon Bias Correlates with Temporal Delays between Ribosome Binding and Translation of New Proteins

**Figure 6:**
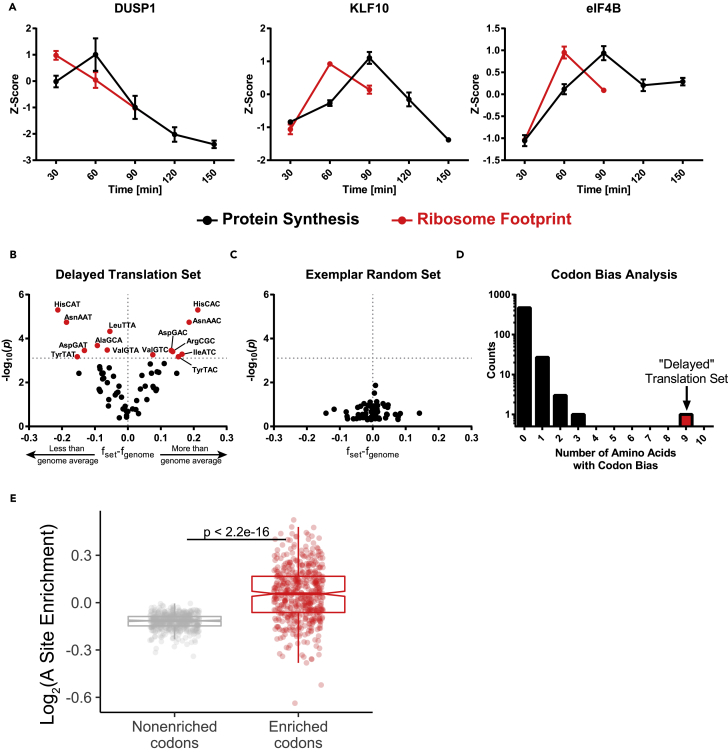
Proteins Exhibiting a Delay between Ribosome Binding and Protein Synthesis Have a Significant Bias in Codon Usage A comparison of RFP analysis and protein synthesis yielded a group of 17 proteins exhibiting a delay between ribosome binding (n = 2) and protein synthesis (n = 3) (A). An analysis of codon usage reveals a statistically significant codon bias present in this set of proteins (B) not present in a randomly generated set of proteins (C). After an analysis of 500 randomly generated protein sets, the extent of this codon bias appears to be unique to these proteins exhibiting a delay between ribosome binding and protein synthesis (D). When considering 500 sets of 100 randomly selected genes, the codons enriched in the set of 17 delayed proteins (Figure 6B, upper right quadrant) occupy the A-site of ribosomes with a higher frequency than other codons (E). Data are mean ± SEM.

Generating time course data for ribosome binding and protein synthesis offered the unique opportunity to characterize the temporal relationships between these processes. Because protein synthesis and ribosome binding occur on scales of different magnitudes, the values were standardized to allow for a direct comparison of the temporal profiles for each. To prevent the analysis of random fluctuations in proteins with unchanging RFP values or protein synthesis rates, only proteins with at least one statistically significant time point (p < 0.05) in both the RFP and MITNCAT datasets were considered, and thus only proteins present in at least two of the four MITNCAT replicates were included in the analysis of temporal delay. These restrictions limited the protein dataset to 90 and the RFP dataset to 400; the overlap between these datasets was 27 proteins. The TE data was compared with MITNCAT data for these 27 proteins. From this analysis, a subset of 17 proteins exhibited a clear delay between ribosome binding and protein synthesis (Figures 6A and S6A and Table S6). One potential cause for this delay is a difference between codon frequency usage in these genes and the corresponding tRNA isoacceptor availability. To determine if these genes exhibit codon usage frequencies that deviate from the genome averages, the average frequency for each codon in each gene (Table S6) was averaged across all the genes in the group. Statistical significance was determined by generating randomized groups of the same size and comparing the codon usage frequency with the genome average. This process was repeated 10^6^ times, and a p value was generated by counting how many randomly generated groups showed a frequency deviation from the genome greater than that of the queried group for each codon. Reported p values were statistically significant if they were less than the Bonferroni corrected *α* = 7.81 × 10^−4^ (accounting for 64 different codons). The cohort of 17 genes exhibiting delayed translation showed a statistically significant codon bias in 14 different codons for 9 different amino acids (Figure 6B). The other 10 genes that did not show delayed translation did not have codon bias compared with the genome average (Figure S6B). To determine if this bias was unique to this group of proteins, 17 random proteins were selected from the whole dataset and queried for codon bias, and this process was repeated 500 times. Of the 500 sets of randomly selected proteins, 469 sets exhibited no statistically significant deviations from the genome average and no sets had a codon bias in more than three amino acids (Figures 6C and 6D). It has been previously reported that longer proteins tend to exhibit a codon bias (Duret and Mouchiroud, 1999), but the distribution of protein length in the set of proteins with delayed translation is not statistically different from that of the entire dataset (Figure S6C).

A majority of the proteins in the delayed translation set share the GO annotation “response to growth factor.” To test whether this codon bias is common among proteins with this GO annotation, we changed the background set from proteins in the MITNCAT dataset to all proteins that have the GO annotation “response to growth factor.” Even after making this change, the proteins exhibiting a delay in translation have a codon bias in nine different amino acids (Figures S6D and S6G) compared with six in the most extreme outlier set drawn from proteins annotated as “response to growth factor” (Figures S6F–S6G). This analysis suggests codon bias is unique to these proteins exhibiting delayed translation and may provide a potential mechanism to explain the temporal discrepancy between ribosome binding and protein synthesis.

If the delay in protein synthesis is indeed due to increased codon bias in these particular transcripts, then ribosomes would be expected to wait longer for a charged tRNA at these codons, and therefore these codons would occupy the A-site of the ribosome with a higher frequency than other codons. To investigate this possibility, we analyzed the frequency of ribosome A-site occupancy for each codon across 500 subsets of 100 randomly selected transcripts from our dataset. When codons were stratified based on whether they were enriched in the 17 proteins with delayed synthesis (Figure 6B, upper right quadrant), we found that these enriched codons occupied the A-site with a greater frequency than other codons across all genes (Figure 6E). This suggests that these particular codons may take longer to get translated, and therefore transcripts enriched for these codons may undergo slower translation, resulting in a delay between ribosome binding and protein synthesis.

## Discussion

Here, we combined BONCAT, pSILAC labeling, and isobaric mass tagging in a novel method, MITNCAT, that enables highly multiplexed quantitative measurements of protein synthesis across multiple time points. Previous studies directly analyzing new protein synthesis have either relied on targeted approaches with pSILAC (Liu et al., 2017) or BONCAT with a limited number of overlapping time points across multiple MS analyses, resulting in poor temporal resolution (Eichelbaum and Krijgsveld, 2014).

One of the critical features of MITNCAT is the improved sensitivity associated with multiplex analysis of pSILAC-labeled peptides from Aha tagged and enriched proteins. On-bead proteolytic digestion of captured proteins and subsequent detection of released peptides eliminates the need to release and detect the Aha-tagged peptide and provides multiple peptides per protein to improve quantification accuracy. Critically, incorporation of pSILAC differentiates newly synthesized proteins from potential non-specifically retained background proteins, improving the stringency of the analysis. Finally, labeling peptides from each condition with isobaric tags enables the multiplexed analysis of many conditions while simultaneously increasing the MS and MS/MS signal intensity by the summation of the isobarically tagged peptides. Overall, combining these techniques into a coherent strategy improved the sensitivity of the method, allowing for decreased labeling times and thus improved temporal resolution, as demonstrated by the 15-min temporal resolution following EGF stimulation. Further improvements to the sensitivity, combined with more stringent washing and increased sample loading, should allow for temporal resolution in the minute time frame.

Application to the UPR demonstrated the reliability of this method to measure translationally controlled changes in protein synthesis. Global down-regulation of translation was observed, a result that was not captured via analysis of RNA abundance. Furthermore, one cluster of proteins was down-regulated to a greater extent than the global average. This cluster contained proteins associated with translational machinery and the glycolysis pathway. The mechanism behind the specific down-regulation of these proteins is not known, but many of these proteins are classified as housekeeping genes that are expressed at high levels across tissue types (Eisenberg and Levanon, 2013). Perhaps because these proteins were expressed at such high levels, global inhibition of translation resulted in a greater degree of down-regulation of these proteins compared with other proteins with lower basal translational rates. We also observed the up-regulation of protein-folding chaperones and other proteins associated with protein degradation and survival consistent with an acute stress response.

Application of MITNCAT to quantify protein synthesis following EGF stimulation enabled us to group proteins based on temporal changes in synthesis. We quantified dynamic synthesis rates for over a thousand proteins and observed IEGs and DEGs to be synthesized simultaneously, peaking between 60 and 90 min post-treatment. LRGs began to be synthesized starting around 90 min and continued to increase in synthesis through 150 min post-treatment. These increases in protein synthesis were matched by observed changes in RNA abundance, suggesting control at the level of transcription. Interestingly, ribosomal proteins and associated translation factors demonstrated increased synthesis as early as 15 min following EGF stimulation, and many of these proteins maintained increased synthesis throughout the time course, yet these changes were not matched by corresponding changes in RNA abundance. The disparity between protein synthesis rates and transcript levels suggested regulation at the level of TE, which was further confirmed by RFP. It is worth noting that the altered synthesis rates of this very large group of proteins could not have been detected by mRNA-seq (transcript expression) alone. RFP would have suggested this increase, but MITNCAT was easily able to detect these changes, including directly measuring increased protein synthesis in the first 15 min following EGF stimulation. Therefore, MITNCAT could also serve as an orthogonal method to validate RFP datasets.

Finally, the comparison of temporal changes in RFP analysis and protein synthesis revealed a class of proteins that demonstrated a temporal delay between ribosome binding and protein synthesis. Analysis of the sequences coding for these proteins revealed a statistically significant bias in their codon usage frequencies that was unique to this group. This correlation opens the possibility for regulation of protein expression based on the availability of specific tRNA isoacceptors and presence of modified nucleosides in tRNAs.

Overall, MITNCAT is broadly applicable to a range of biological systems, provides synthesis rate information for thousands of proteins in a high-throughput, discovery-based approach, and yet can also be coupled to targeted MS-based approaches to quantify temporal dynamics of protein synthesis for *a priori* selected proteins. The sensitivity of MITNCAT provides high temporal resolution, and application of this approach led to the identification of many proteins whose synthesis was significantly altered as rapidly as 15 min following stimulation. Future application of this approach to other biological systems will provide novel insights into the regulation between transcription and translation.

### Limitations of the Study

To efficiently label newly synthesized proteins with SILAC and Aha labels, cells must be subjected to a 30-min starvation of arginine, lysine, and methionine. This amino acid starvation may trigger a stress response in the cells. In the absence of proper controls, this may result in artificially high expression of stress proteins. In this study, all data points are normalized to untreated controls to normalize out these effects. However, when examining protein synthesis during the UPR using MITNCAT, increased stress response proteins in the negative controls may normalize out a portion of the response that would have occurred in response to the UPR. This limitation should be considered when using MITNCAT for studying stress responses.

## Methods

All methods can be found in the accompanying Transparent Methods supplemental file.

## Data and Software Availability

All methods can be found in the accompanying Transparent Methods supplemental file. The accession number for the ribosome footprint and transcript sequencing data reported in this paper is NCBI BioProject: PRJNA478455. The accession number for the proteomics data reported in this paper is ProteomeXchange Consortium (PRIDE): PXD009592.

## Supplemental Information

Document S1. Transparent Methods and Figures S1–S6

Table S1. Aha Time Course, Related to Figure 1

Table S2. Tunicamycin Time Course, Related to Figure 2

Table S3. EGF 30 min Time Course, Related to Figure 3

Table S4. EGF 15 min Time Course, Related to Figure 4

Table S5. RNASeq, Ribosome Footprinting, Transcriptional Efficiency, Related to Figure 5

Table S6. Codon Bias, Related to Figure 6

## References

[bib1] Amit I., Citri A., Shay T., Lu Y., Katz M., Zhang F., Tarcic G., Siwak D., Lahad J., Jacob-Hirsch J. (2007). A module of negative feedback regulators defines growth factor signaling. Nat. Genet..

[bib2] Avraham R., Sas-Chen A., Manor O., Steinfeld I., Shalgi R., Tarcic G., Bossel N., Zeisel A., Amit I., Zwang Y. (2010). EGF decreases the abundance of MicroRNAs that restrain oncogenic transcription factors. Sci. Signal..

[bib3] Avraham R., Yarden Y. (2011). Feedback regulation of EGFR signalling: decision making by early and delayed loops. Nat. Rev. Mol. Cell Biol..

[bib4] Berlanga J.J., Santoyo J., De Haro C. (1999). Characterization of a mammalian homolog of the GCN2 eukaryotic initiation factor 2alpha kinase. Eur. J. Biochem..

[bib5] Bowling H., Bhattacharya A., Zhang G., Lebowitz J.Z., Alam D., Smith P.T., Kirshenbaum K., Neubert T.A., Vogel C., Chao M.V., Klann E. (2016). BONLAC: a combinatorial proteomic technique to measure stimulus-induced translational profiles in brain slices. Neuropharmacology.

[bib6] Chan C.T.Y., Dyavaiah M., DeMott M.S., Taghizadeh K., Dedon P.C., Begley T.J. (2010). A quantitative systems approach reveals dynamic control of tRNA modifications during cellular stress. PLoS Genet..

[bib7] Chan C.T.Y., Pang Y.L.J., Deng W., Babu I.R., Dyavaiah M., Begley T.J., Dedon P.C. (2012). Reprogramming of tRNA modifications controls the oxidative stress response by codon-biased translation of proteins. Nat. Commun..

[bib8] Chen J.J., London I.M. (1995). Regulation of protein synthesis by heme-regulated eIF-2 alpha kinase. Trends Biochem. Sci..

[bib9] Chionh Y.H., McBee M., Babu I.R., Hia F., Lin W., Zhao W., Cao J., Dziergowska A., Malkiewicz A., Begley T.J. (2016). tRNA-mediated codon-biased translation in mycobacterial hypoxic persistence. Nat. Commun..

[bib10] Dieterich D.C., Link A.J., Graumann J., Tirrell D.A., Schuman E.M. (2006). Selective identification of newly synthesized proteins in mammalian cells using bioorthogonal noncanonical amino acid tagging (BONCAT). Proc. Natl. Acad. Sci. U S A.

[bib11] Doherty M.K., Whitehead C., McCormack H., Gaskell S.J., Beynon R.J. (2005). Proteome dynamics in complex organisms: using stable isotopes to monitor individual protein turnover rates. Proteomics.

[bib12] Duret L., Mouchiroud D. (1999). Expression pattern and, surprisingly, gene length shape codon usage in Caenorhabditis, Drosophila, and Arabidopsis. Proc. Natl. Acad. Sci. U S A.

[bib13] Eichelbaum K., Krijgsveld J. (2014). Rapid temporal dynamics of transcription, protein synthesis, and secretion during macrophage activation. Mol. Cell. Proteomics.

[bib14] Eichelbaum K., Winter M., Diaz M.B., Herzig S., Krijgsveld J. (2012). Selective enrichment of newly synthesized proteins for quantitative secretome analysis. Nat. Biotechnol..

[bib15] Eisenberg E., Levanon E.Y. (2013). Human housekeeping genes, revisited. Trends Genet..

[bib16] Feldman M.E., Yarden Y. (2014). Steering tumor progression through the transcriptional response to growth factors and stroma. FEBS Lett..

[bib17] Feng G.S., Chong K., Kumar A., Williams B.R. (1992). Identification of double-stranded RNA-binding domains in the interferon-induced double-stranded RNA-activated p68 kinase. Proc. Natl. Acad. Sci. U S A.

[bib18] Filbin M.E., Kieft J.S. (2009). Toward a structural understanding of IRES RNA function. Curr. Opin. Struct. Biol..

[bib19] Gingras A.-C., Raught B., Sonenberg N. (2001). Regulation of translation initiation by FRAP/mTOR. Genes Dev..

[bib20] Golan-Lavi R., Giacomelli C., Fuks G., Zeisel A., Sonntag J., Sinha S., Köstler W., Wiemann S., Korf U., Yarden Y., Domany E. (2017). Coordinated pulses of mRNA and of protein translation or degradation produce EGF-induced protein bursts. Cell Rep..

[bib21] Howden A.J., Geoghegan V., Katsch K., Efstathiou G., Bhushan B., Boutureira O., Thomas B., Trudgian D.C., Kessler B.M., Dieterich D.C. (2013). QuaNCAT: quantitating proteome dynamics in primary cells. Nat. Methods.

[bib22] Ingolia N.T. (2016). Ribosome footprint profiling of translation throughout the genome. Cell.

[bib23] Ingolia N.T., Ghaemmaghami S., Newman J.R.S., Weissman J.S. (2009). Genome-wide analysis in vivo of translation with nucleotide resolution using ribosome profiling. Science.

[bib24] Iwasaki S., Ingolia N.T. (2017). The growing toolbox for protein synthesis studies. Trends Biochem. Sci..

[bib25] Jovanovic M., Rooney M.S., Mertins P., Przybylski D., Chevrier N., Satija R., Rodriguez E.H., Fields A.P., Schwartz S., Raychowdhury R. (2015). Immunogenetics. Dynamic profiling of the protein life cycle in response to pathogens. Science.

[bib26] Kiick K.L., Saxon E., Tirrell D.A., Bertozzi C.R. (2002). Incorporation of azides into recombinant proteins for chemoselective modification by the Staudinger ligation. Proc. Natl. Acad. Sci. U S A.

[bib27] Liu T.Y., Huang H.H., Wheeler D., Xu Y., Wells J.A., Song Y.S., Wiita A.P. (2017). Time-resolved proteomics extends ribosome profiling-based measurements of protein synthesis dynamics. Cell Syst..

[bib28] Ma Y., McClatchy D.B., Barkallah S., Wood W.W., Yates J.R. (2017). HILAQ: a novel strategy for newly synthesized protein quantification. J. Proteome Res..

[bib29] McShane E., Sin C., Zauber H., Wells J.N., Donnelly N., Wang X., Hou J., Chen W., Storchova Z., Marsh J.A. (2016). Kinetic analysis of protein stability reveals age-dependent degradation. Cell.

[bib30] Novoa I., Zhang Y., Zeng H., Jungreis R., Harding H.P., Ron D. (2003). Stress-induced gene expression requires programmed recovery from translational repression. EMBO J..

[bib31] Reddy R.J., Gajadhar A.S., Swenson E.J., Rothenberg D.A., Curran T.G., White F.M. (2016). Early signaling dynamics of the epidermal growth factor receptor. Proc. Natl. Acad. Sci. U S A.

[bib32] Rowlands A.G., Montine K.S., Henshaw E.C., Panniers R. (1988). Physiological stresses inhibit guanine-nucleotide-exchange factor in Ehrlich cells. Eur. J. Biochem..

[bib33] Savitski M.M., Mathieson T., Zinn N., Sweetman G., Doce C., Becher I., Pachl F., Kuster B., Bantscheff M. (2013). Measuring and managing ratio compression for accurate iTRAQ/TMT quantification. J. Proteome Res..

[bib34] Schwanhäusser B., Busse D., Li N., Dittmar G., Schuchhardt J., Wolf J., Chen W., Selbach M. (2011). Global quantification of mammalian gene expression control. Nature.

[bib35] Schwanhäusser B., Gossen M., Dittmar G., Selbach M. (2009). Global analysis of cellular protein translation by pulsed SILAC. Proteomics.

[bib36] Sears R., Nuckolls F., Haura E., Taya Y., Tamai K., Nevins J.R. (2000). Multiple Ras-dependent phosphorylation pathways regulate Myc protein stability. Genes Dev..

[bib37] Shi T., Niepel M., McDermott J.E., Gao Y., Nicora C.D., Chrisler W.B., Markillie L.M., Petyuk V.A., Smith R.D., Rodland K.D. (2016). Conservation of protein abundance patterns reveals the regulatory architecture of the EGFR-MAPK pathway. Sci. Signal..

[bib38] Stefanovsky V.Y., Pelletier G., Hannan R., Gagnon-Kugler T., Rothblum L.I., Moss T. (2001). An immediate response of ribosomal transcription to growth factor stimulation in mammals is mediated by ERK phosphorylation of UBF. Mol. Cell.

[bib39] Thompson A., Schäfer J., Kuhn K., Kienle S., Schwarz J., Schmidt G., Neumann T., Hamon C. (2003). Tandem mass tags: a novel quantification strategy for comparative analysis of complex protein mixtures by MS/MS. Anal. Chem..

[bib40] Wang X., Eno C.O., Altman B.J., Zhu Y., Zhao G., Olberding K.E., Rathmell J.C., Li C. (2011). ER stress modulates cellular metabolism. Biochem. J..

[bib41] Waters K.M., Liu T., Quesenberry R.D., Willse A.R., Bandyopadhyay S., Kathmann L.E., Weber T.J., Smith R.D., Wiley H.S., Thrall B.D. (2012). Network analysis of epidermal growth factor signaling using integrated genomic, proteomic and phosphorylation data. PLoS One.

[bib42] Welle K.A., Zhang T., Hryhorenko J.R., Shen S., Qu J., Ghaemmaghami S. (2016). Time-resolved analysis of proteome dynamics by tandem mass tags and stable isotope labeling in cell culture (TMT-SILAC) hyperplexing. Mol. Cell. Proteomics.

[bib43] Zhang Y., Wolf-yadlin A., Ross P.L., Pappin D.J., Rush J., Lauffenburger D.A., White F.M. (2005). Time-resolved mass spectrometry of tyrosine phosphorylation sites in the epidermal growth factor receptor signaling network reveals dynamic modules. Mol. Cell. Proteomics.

