# Supplementary material for: A Proteomics Approach to Profiling the Temporal Translational Response to Stress and Growth
Source: iScience. 2018 Nov 5;9:367–81. doi: 10.1016/j.isci.2018.11.004 (PMC6249402; doi:10.1016/j.isci.2018.11.004)
Supplement: Document S1. Transparent Methods and Figures S1–S6 [file mmc1.pdf]

ISCI, Volume *9*

Supplemental Information

A Proteomics Approach to Profiling the Temporal Translational Response to Stress and Growth

Daniel A. Rothenberg, J. Matthew Taliaferro, Sabrina M. Huber, Thomas J. Begley, Peter C. Dedon, and Forest M. White

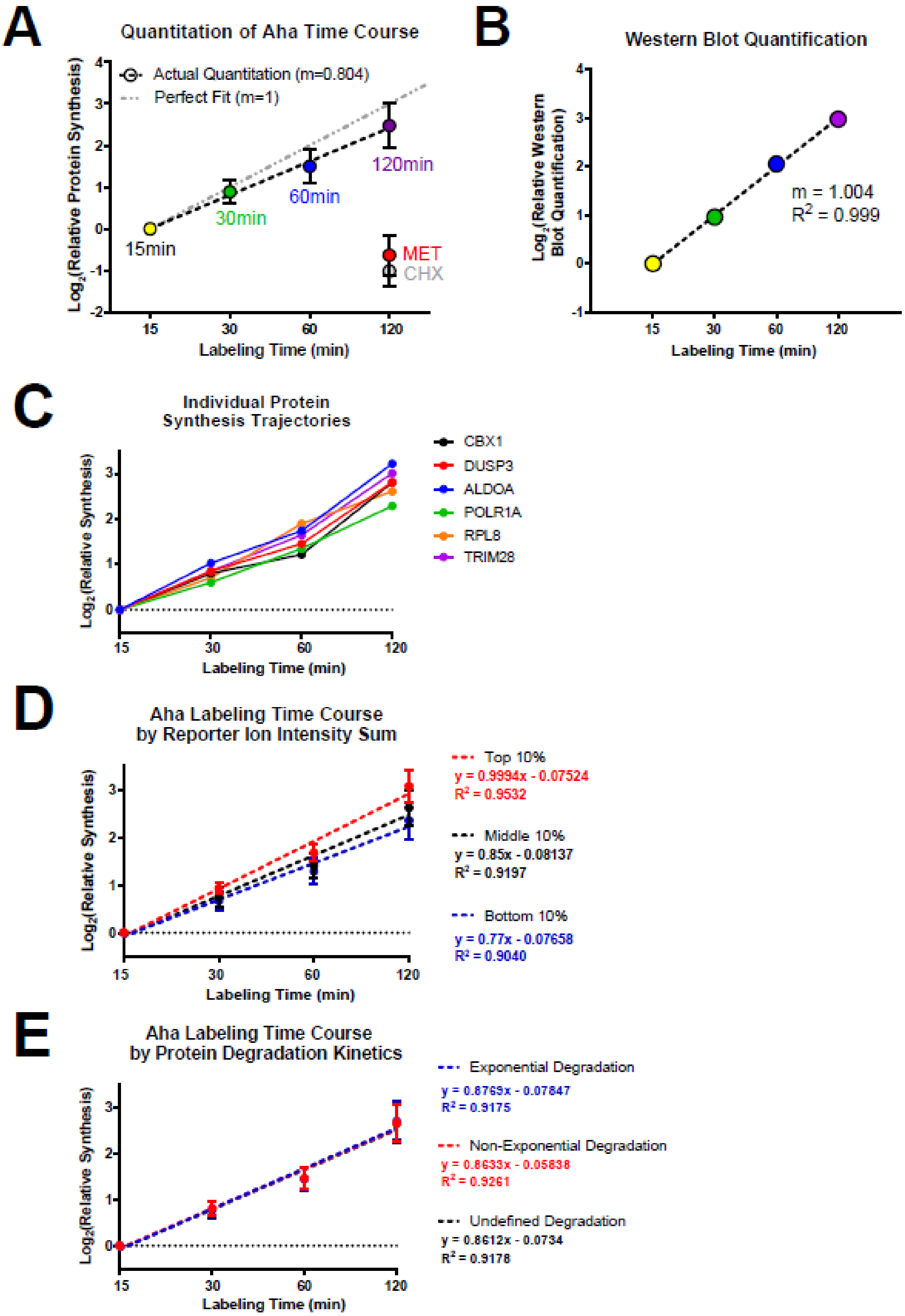
A
Quantitation of Aha Time Course
Actual Quantitation (m=0.804)
Perfect Fit (m=1)
Log2(Relative Protein Synthesis)
15min
30min
60min
120min
MET
CHX
Labeling Time (min)
B
Western Blot Quantification
Log2(Relative Western Blot Quantification)
m = 1.004
R2 = 0.999
Labeling Time (min)
C
Individual Protein Synthesis Trajectories
CBX1
DUSP3
ALDOA
POLR1A
RPL8
TRIM28
Log2(Relative Synthesis)
Labeling Time (min)
D
Aha Labeling Time Course by Reporter Ion Intensity Sum
Top 10%
y = 0.9994x - 0.07524
R2 = 0.9532
Middle 10%
y = 0.85x - 0.08137
R2 = 0.9197
Bottom 10%
y = 0.77x - 0.07658
R2 = 0.9040
Log2(Relative Synthesis)
Labeling Time (min)
E
Aha Labeling Time Course by Protein Degradation Kinetics
Exponential Degradation
y = 0.8769x - 0.07847
R2 = 0.9175
Non-Exponential Degradation
y = 0.8633x - 0.05838
R2 = 0.9261
Undefined Degradation
y = 0.8612x - 0.0734
R2 = 0.9178
Log2(Relative Synthesis)
Labeling Time (min)

Figure S1. Analysis of Aha labeling time course shows reliable quantitation of newly-synthesized proteins across replicates, related to Figure 1. A replicate of the time course in HeLa cells demonstrates excellent quantitation independent of cell line (A). Quantitation of the western blot from Figure 1D demonstrates a linear 1-to-1 relationship between labeling time and labeling intensity (B). A selection of individual protein trajectories reveals a robust labeling time-dependent increase in enrichment (C). Proteins with increased reporter ion abundance have a stronger 1:1 correlation with Aha-labeling time, but even the bottom 10% of proteins by ion intensity have a correlation of over 75% (D). The relationship between labeling time and protein abundance is not impacted by varying protein degradation kinetics characterized in [36] (E). Data are medians +/- SEM.

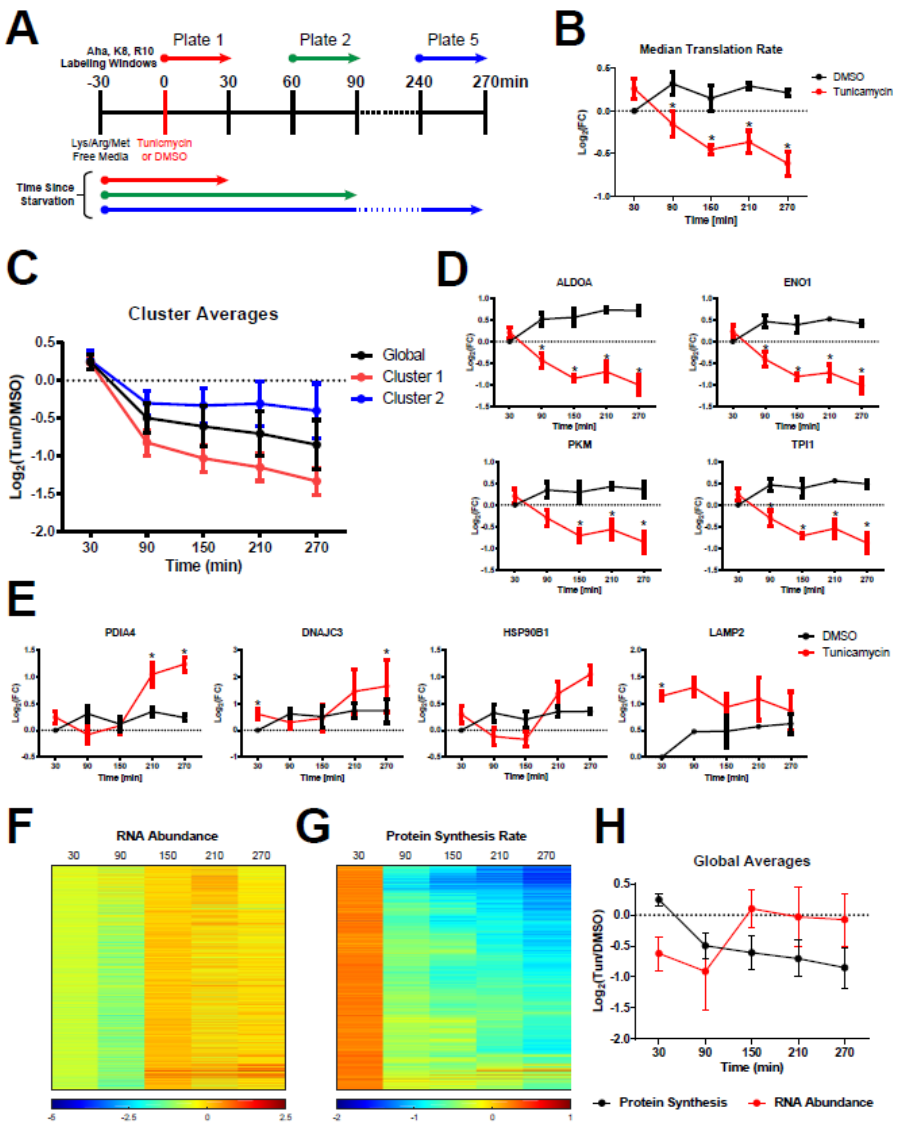

Figure S2. Temporal profiles of protein synthesis during the unfolded protein response following tunicamycin treatment, related to Figure 2. Newly synthesized proteins were labeled with Aha and SILAC amino acids for 30 minutes every hour following tunicamycin treatment, with matched negative controls to account for changes due to starvation (A). Tunicamycin treatment results in a decrease in global protein synthesis (B), with one cluster of proteins showing a decrease below the median and one cluster showing an increase above the median (C). Proteins associated with glycolysis are in cluster 1, demonstrating especially strong down-regulation (D), whereas other stress response proteins are up-regulated following tunicamycin treatment (E). Measurement of RNA abundance by mRNA-Seq (F) does not capture the global decrease in protein synthesis as measured by BONCAT (G,H). n=3 biological replicates for MS data, n=1 for mRNA-Seq. Data are mean +/- SEM. * = $p < 0.05$

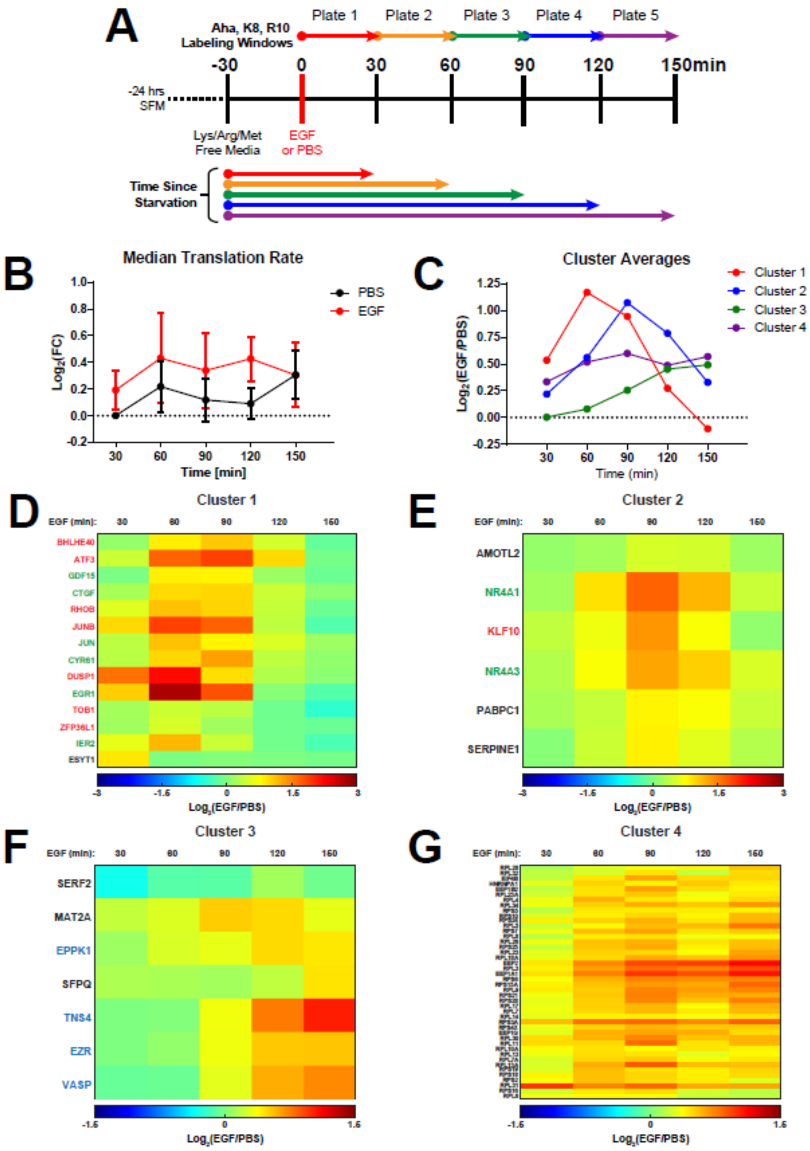

Figure S3. Changes in protein synthesis following EGF stimulation were clustered into four groups based on temporal behavior, related to Figure 3. Newly synthesized proteins were labeled with Aha and SILAC amino acids in consecutive 30 minute windows following EGF treatment, with matched negative controls to account for changes due to starvation (A). EGF treatment does not result in a statistically significant change in global translation (B). Each protein cluster demonstrates a unique temporal profile in response to EGF treatment (C). Clusters 1 and 2 are transiently expressed proteins and contain IEGs (green) and DEGs (red) (D,E), whereas cluster 3 is up-regulated at a later time and consists of LRGs (blue) (F). Cluster 4 contains primarily proteins associated with translational machinery, and is up-regulated across all time points sampled (G). n=4 biological replicates. Data are mean +/- SEM. * = $p < 0.05$

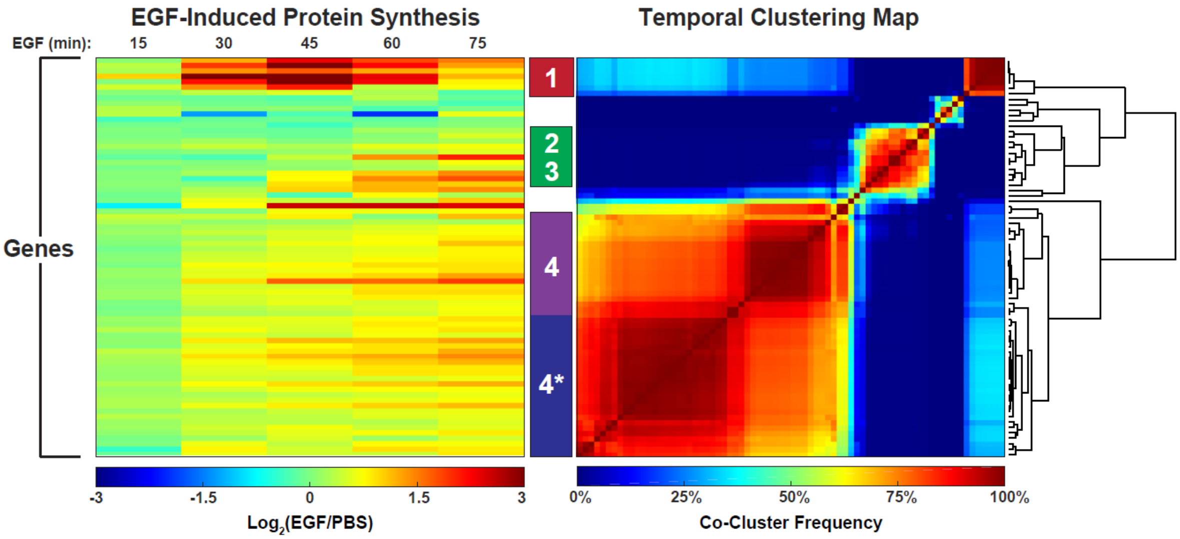

Figure S4. Decreasing the duration of Aha labeling increased temporal resolution and yielded new insights into protein synthesis dynamics, related to Figure 4. Temporal changes in protein synthesis were sampled every 15 minutes over the first 75 minutes, and clustered by k-means clustering. n=3 biological replicates.

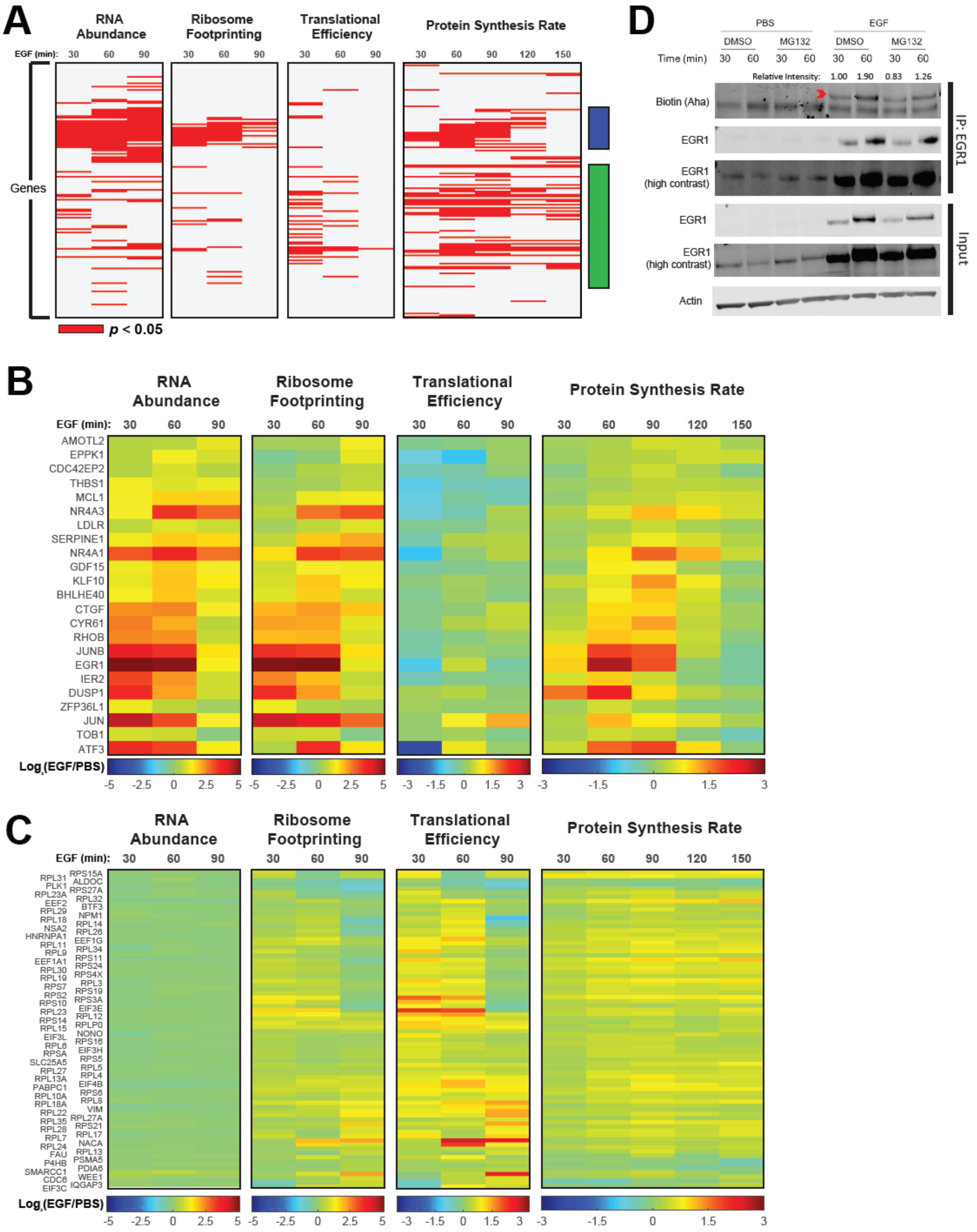
A
RNA Abundance
Ribosome Footprinting
Translational Efficiency
Protein Synthesis Rate
EGF (min): 30 60 90 30 60 90 30 60 90 30 60 90 120 150
Genes
p < 0.05
D
PBS
EGF
DMSO
MG132
Time (min) 30 60 30 60 30 60 30 60
Relative Intensity: 1.00 1.90 0.83 1.26
Biotin (Aha)
EGR1
EGR1 (high contrast)
IP: EGR1
Input
Actin
B
EGF (min):
AMOTL2
EPPK1
CDC42EP2
THBS1
MCL1
NR4A3
LDLR
SERPINE1
NR4A1
GDF15
KLF10
BHLHE40
CTGF
CYR61
RHOB
JUNB
EGR1
IER2
DUSP1
ZFP36L1
JUN
TOB1
ATF3
Log(EGF/PBS)
-5 -2.5 0 2.5 5
-3 -1.5 0 1.5 3
C
RPL31 RPS15A PLK1 ALDOC RPL23A RPS27A EEF2 RPL32 RPL29 BTF3 RPL18 NPM1 NSA2 RPL14 HNRNPA1 RPL26 RPL11 EEF1G RPL9 RPL34 EEF1A1 RPS11 RPL30 RPS24 RPL19 RPS4X RPS7 RPL3 RPS2 RPS19 RPS10 RPS3A RPL23 EIF3E RPS14 RPL12 RPL15 RPLP0 EIF3L NONO RPL6 RPS16 RPSA EIF3H SLC25A5 RPS5 RPL27 RPL5 RPL13A RPL4 PABPC1 EIF4B RPL10A RPS6 RPL18A RPL8 RPL22 VIM RPL35 RPL27A RPL28 RPS21 RPL7 RPL17 RPL24 NACA FAU RPL13 P4HB PSMA5 SMARCC1 PDIA6 CDC6 WEE1 EIF3C IQGAP3

Figure S5. Temporal response to EGF stimulation compared between transcript expression, ribosome binding, and protein synthesis, related to Figure 5. Statistical significance of the network-wide temporal response to EGF stimulation was assessed at the transcript expression level by mRNA-Seq (n=2), while translation rates were estimated by RFP (n=2), and protein synthesis rates measured by MITNCAT (n=3) (A). Comparing these datasets revealed a set of proteins whose altered synthesis correlated with changes in RNA abundance (blue bar) (B), suggesting regulation at transcription. Another group demonstrated changes in synthesis correlating with changes in translational efficiency (green bar) (C), suggesting regulation at translation. Measuring protein synthesis of Aha-labeled EGR1 by immunoprecipitation followed by click-labeling yielded relative protein synthesis changes in agreement with MITNCAT data. Blocking protein degradation by co-treating with proteasome inhibitor MG132 did not alter the synthesis profile of EGR1 (D).

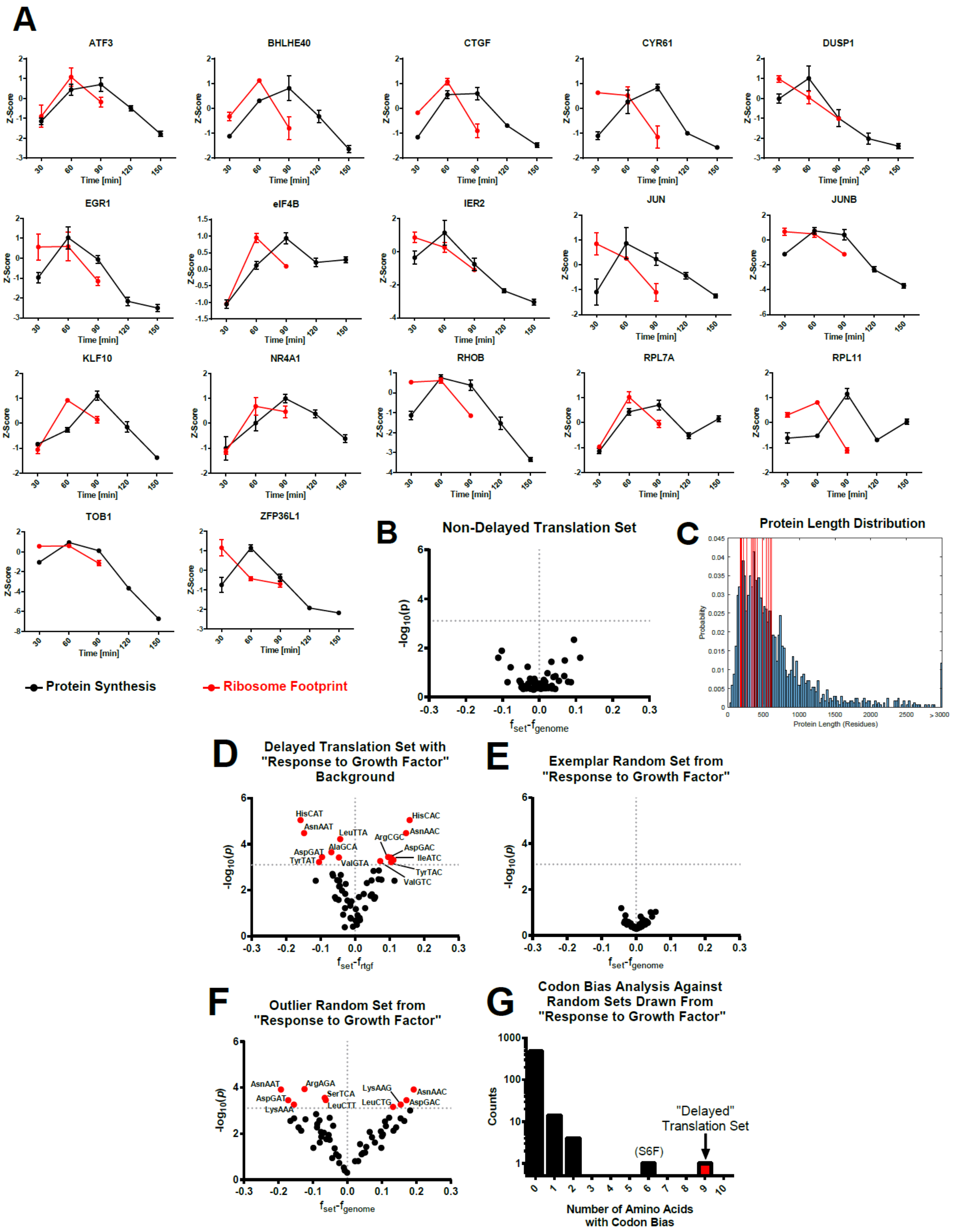

A
ATF3
BHLHE40
CTGF
CYR61
DUSP1
EGR1
eIF4B
IER2
JUN
JUNB
KLF10
NR4A1
RHOB
RPL7A
RPL11
TOB1
ZFP36L1
Z-Score
Time [min]
Protein Synthesis
Ribosome Footprint
B
Non-Delayed Translation Set
-log10(p)
fset-fgenome
C
Protein Length Distribution
Probability
Protein Length (Residues)
D
Delayed Translation Set with
"Response to Growth Factor"
Background
HisCAT
AsnAAT
LeuTTA
ArgCGC
HisCAC
AsnAAC
AspGAC
IleATC
TyrTAC
ValGTC
AspGAT
TyrTAT
AlaGCA
ValGTA
fset-frtgf
E
Exemplar Random Set from
"Response to Growth Factor"
F
Outlier Random Set from
"Response to Growth Factor"
AsnAAT
ArgAGA
SerTCA
LysAAG
AsnAAC
AspGAT
LysAAA
LeuCTT
LeuCTG
AspGAC
G
Codon Bias Analysis Against
Random Sets Drawn From
"Response to Growth Factor"
Counts
"Delayed"
Translation Set
(S6F)
Number of Amino Acids
with Codon Bias

Figure S6. Proteins exhibiting a delay between ribosome binding and protein synthesis have a significant bias in codon usage, related to Figure 6. A comparison of RFP analysis (n=2) and protein synthesis (n=3) yielded a group of 17 proteins exhibiting a delay between ribosome binding and protein synthesis (A). The other 10 proteins that in this dataset that did not exhibit a delay also did not demonstrate a codon bias (B). Analysis of protein length reveals that the proteins exhibiting delayed translation (red lines) do not differ in length from the rest of the proteins in the dataset (C). Changing the background from all proteins in our dataset to all proteins with the GO annotation “response to growth factor” (RTGF) did not affect the number of amino acids with codon bias in the delayed protein set (D) compared to randomly selected groups of 17 proteins from that background (E-G). Data are mean +/- SEM.

Transparent Methods

Cell Culture

For Aha time course and UPR experiments, MCF10a cells (courtesy of Joan Brugge, Harvard Medical School) were cultured in DMEM:F12 media supplemented with 5% horse serum, 20 ng/mL EGF, 500 µg/mL hydrocortisone, 100 ng/mL cholera toxin, 10 µg/mL insulin, 1% penicillin/streptomycin, and 2 mM glutamine. Cells were passaged every third day at a 1:4 ratio, and all experiments were performed on the third day following passaging. For EGF stimulation experiments, HeLa cells (from ATCC) were cultured in DMEM supplemented with 10% fetal bovine serum (FBS), 1% penicillin/streptomycin, and 2 mM glutamine. Cells were passaged every 3 days and split between 1:4 and 1:6 ratio, and serum starvation was initiated on the second day following passaging.

Western Blot Quantitation

Protein concentration of lysates were measured via bicinchroninic acid (BCA) assays and normalized to 1 mg/mL. For measurements of Aha incorporation, DBCO-biotin was added to the lysates for 1 hour at a concentration of 25 µM. The reaction was quenched by adding sodium azide to roughly 100 mM. LDS buffer was added to 1x, and beta-mercaptoethanol was added to 1%. Samples were loaded onto a NuPAGE Novex 4-12% Bis-Tris Midi Protein Gels (Invitrogen) and run at 160V for 1 hour. Samples were transferred to a nitrocellulose membrane at 100V for 1 hour. Membranes were blocked with LICOR PBS blocking buffer for 30 minutes at room temperature. Biotinylated proteins were probed with IRDye 680RD-conjugated streptavidin (LICOR #32230). The following primary antibodies were used: GAPDH 1:10,000 (Cell Signaling #5174). The following secondary antibodies were used: IRDye 800CW-conjugated goat-anti-rabbit 1:10,000 (LICOR # 926-32211) and IRDye 680LT-conjugated goat-anti-mouse 1:10,000 (LICOR #926-68031). Western blots were imaged on a LICOR Odyssey instrument.

Quantitation of the Western blot was performed using ImageJ software (https://imagej.nih.gov/ij). The average signal intensity of each lane was measured starting at a molecular weight below endogenously biotinylated proteins [1], normalized to the methionine control lane, and log2 transformed.

Metabolic Labeling and Cell Lysis

For all EGF experiments, cells were changed to serum-free media 24 hours before stimulation. In all experiments, growth media was removed and replaced with lysine/arginine/methionine (KRM)-free DMEM:F12 media 30 minutes prior to time course initiation. For EGF experiments, EGF was added to the media to achieve a final concentration of 20 ng/mL or an equivalent volume of PBS was added as a control. For UPR experiments, tunicamycin was added to the media to achieve a final concentration of 10 µg/mL or an equal volume of DMSO was added as a control. At the appropriate time points following treatment, Aha was added to the media to achieve a final concentration of 3 mM, and $^{15}N_4{}^{13}C_6$ arginine (R10) and $^{15}N_2{}^{13}C_6$ lysine (K8) were added to 0.5 mM. After 30 minutes of Aha/K8/R10 labeling, the media was aspirated, and the cells were washed in ice cold PBS supplemented with 300 µg/mL cycloheximide (CHX). Cells were lysed in 1% SDS in PBS supplemented with 50 mM N-ethylmaleimide (NEM) and 300 µg/mL CHX. 1.5 mL of -20°C acetone was added to each tube immediately following lysis to precipitate proteins. Proteins were precipitated at -20°C for at least an hour and up to overnight.

Sample Processing and Fractionation

Following precipitation, the samples were centrifuged at maximum speed (~21,000 x g) for 15 minutes at room temperature to pellet the precipitated proteins. The supernatant was aspirated off the pellet, and the pellet was allowed to air dry for 15 minutes to evaporate residual acetone. 250 µL of 1% SDS in PBS supplemented with 50 mM NEM was added to the pellet,

along with ~1/10th of a vial of 0.7 mm garnet homogenizing beads to aid in pellet disruption. Samples were alternately vortexed and centrifuged at maximum speed until the pellet was completely solubilized.

Following resuspension, protein concentration was measured via BCA assay, and total protein was normalized to 100-300 µg in 500 µL of 1% SDS in PBS. Each sample was diluted 2-fold in 8 M urea + 850 mM NaCl. 12.5 µL of the lysate was aliquoted for biotin labeling of Aha-labeled proteins and western blot analysis. The remaining volume for each sample was applied to 30 µL of DBCO-agarose beads that had pre-equilibrated by washing 3x in 1 mL 0.8% SDS in PBS. Click enrichment took place overnight at room temperature on a rotor.

After overnight click incubation, the bead/supernatant mixture was transferred to an empty spin column and allowed to drain into Eppendorf tubes to collect the supernatant. 12.5 µL of supernatant was aliquoted for biotin labeling of Aha-labeled proteins and western blot analysis. The tubes were rinsed out with 1 mL MilliQ water and added to the spin column, allowing to drain into a waste container. To reduce disulfide bridges, 1 mL 10 mM DTT in 0.8% SDS in PBS was added to the columns and the columns were capped and placed on a rotor at 50°C. Following reduction, the column was drained, and 1 mL 50 mM NEM in 0.8% SDS in PBS was added to the column to alkylate the newly reduced cysteines. The columns were placed on a rotor at room temperature for 30 minutes. The column was once again drained, and the beads were washed 8x with 1 mL 0.8% SDS in PBS, 8x with 1 mL 8 M urea, and 8x with 1 mL 20% acetonitrile (MeCN). After the second wash in each step, the column was capped and allowed to stand for 10 minutes.

Following the final wash step, the beads were completely dried by spinning the excess wash buffer into an empty Eppendorf tube. The beads were resuspended in 300 µL of digest buffer (200 mM triethyl ammonium bicarbonate (TEAB) + 10% MeCN) and transferred to a fresh tube. The column was twice rinsed with 300 µL digest buffer, with the rinses being combined with the sample. The beads were pelleted by centrifuging at 5000 x g for 5 minutes at room

temperature. The supernatant was carefully removed and replaced with 100 µL 1 ng/µL trypsin in digest buffer. The on-bead digest proceeded overnight at room temperature on a rotor.

Following the overnight digest, an aliquot of TMT dissolved in 30 µL anhydrous MeCN was added directly to the tube (beads included). TMT labeling proceeded for 1 hour at room temperature, after which the reaction was quenched with the addition of 15 µL 1 M Tris pH 7.4. The volumes were reduced to about 50 µL in a vacuum centrifuge. All samples (including beads) were combined into a single Eppendorf tube. The individual tubes were rinsed 3x with 40 µL 50% MeCN + 0.1% AcOH, with the rinses being combined with the pooled sample. The sample was completely dried in a vacuum centrifuge.

Following drying, the sample (beads included) was resuspended in 500 µL 10 mM TEAB pH 8. The fritted end of a 200 µm i.d. capillary was placed in the bead pack, and the sample was loaded onto a ZORBAX Extend 300 C18 column (Agilent #770995-902) at 750 psi. The C18 column was placed in line with an HPLC, and the following gradient was run at a flow rate of 1mL/min. (A = 10mM TEAB pH 8, B = 99% MeCN, 10mM TEAB): 0-5 min, 0-5% B; 5-50 min, 5-40% B; 50-59 min, 40-70% B; 59-64 min, 70% B; 64-65 min, 70 to 1% B. Fractions were collected every minute between 5 min and 65 min. Every 12th fraction was concatenated together to give 12 total samples (5 fractions per sample). Samples were placed into a vacuum centrifuge overnight or until the sample reached dryness.

Mass Spectrometry Analysis

Dried samples were resuspended in 50 µL 0.1% formic acid (FA). The samples were placed in a ThermoFisher Easy nLC 1000 autosampler and analyzed on a ThermoFisher QExactive Plus mass spectrometer using 25 µL of the resuspended sample. The sample was analyzed using the following gradient over a C18 column (A = 0.1% FA, B = 80% MeCN in 0.1% FA): 0-4 min, 0-14% B; 4-50 min, 13-42% B; 50-57 min, 42-60% B; 57-60 min, 60-100% B; 60-68 min, 100% B; 68-69 min, 100-0% B; 69-75 min, 0% B. The instrument was operated in data

dependent acquisition mode, with the top 15 most abundant precursors with charge of +2 or greater selected for fragmentation and dynamic exclusion set to 15 s. Precursors were isolated with a window of 0.4 *m/z* and fragmented via HCD at 33 NCE. Precursor scan settings were set to AGC = 3e6, maximum IT = 50 ms, and resolution of 70,000. MS2 scan settings were set to AGC = 1e5, maximum IT = 300 ms, and resolution of 35,000. The total acquisition time was 75 minutes per sample.

MS data files were searched on MASCOT version 2.4 with fixed modifications for NEM alkylation on cysteines (+125.047 Da), addition of TMT 6-plex to N-termini and lysine residues (+229.163 Da). Variable modifications were SILAC R10 on arginine residues (+10.008 Da), SILAC K8 on lysine residues (+8.014 Da), addition of TMT 6-plex to SILAC K8 lysine residues (+237.177 Da), Aha substitution for methionine residues (-4.986 Da), diaminobutyrate (reduced Aha) substitution for methionine residues (-30.976 Da), oxidation of methionine residues (+15.995 Da) and phosphorylation on tyrosine, threonine, and serine residues (+79.966 Da). Precursor tolerance was 10 ppm, fragment tolerance was 15 mmu, two missed cleavages were allowed, and the enzyme was set to trypsin. Peptides were considered to be positively identified if they had a score of at least 25 and newly translated if they contained a SILAC or Aha residue. Peptides with TMT reporter ion intensities less than 1000 in any one channel were discarded. To control for technical variation between channels, in the absence of a statistically significant change in global protein synthesis rate across replicates, values were normalized to the median of each channel within each replicate. Therefore, the EGF dataset was median normalized, but the tunicamycin dataset was not. The mass spectrometry proteomics data have been deposited to the ProteomeXchange Consortium via the PRIDE [[2]] partner repository with the dataset identifier PXD009592.

RNA Sequencing and Ribosome Footprint Analysis

Cells were subjected to identical KRM-free media pretreatment, Aha/K/R labeling, and

PBS+CHX washing conditions as described previously to account for any effects that may be caused by these treatments. Cells were lysed and processed using the Illumina TruSeq Ribo Profile kit (Illumina #RPHMR12126) according to the manufacturer's protocol. RFP samples were sequenced on an Illumina NextSeq instrument with 50 nt single-end reads and 6 nt barcodes with 6 samples per lane. Adapter sequences were removed from the 3' end using Cutadapt. Reads were then mapped to hg38 rRNA sequences using STAR. Reads that aligned to rRNA sequences were removed. The remaining reads were then mapped to an hg38 annotation (Gencode release 26). Reads that uniquely map to this annotation were then quantified using Salmon and a FASTA file containing all hg38 cDNA sequences to generate transcripts per million (TPM) values and counts. A library for total RNA was prepared using the Illumina NeoPrep System and sequenced on an Illumina NextSeq instrument with 40 nt paired-end reads and 6 nt barcodes with 12 samples per lane. For analyses requiring alignment, these reads were mapped to the same hg38 (Gencode release 26) annotation. Quantification was done using Salmon and a FASTA file containing all hg38 cDNA sequences to generate TPM and count values. Only ribosome footprints that mapped to coding regions were considered for quantification and subsequent analyses. Ribosome profiling TE changes and associated p-values were calculated using the Salmon-derived count data and the Xtail package [3]. All ribosome footprint and transcript sequencing data have been deposited to NCBI's BioProject database under accession number PRJNA478455 (https://www.ncbi.nlm.nih.gov/bioproject/478455).

Self-Organizing Map (SOM) Clustering Analysis

A self-organizing map (SOM) was used to cluster proteins from UPR experiments that exhibited similar protein synthesis dynamics following tunicamycin treatment. Clustering analysis was performed using the Self Organizing Map Toolbox MATLAB package (http://www.cis.hut.fi/projects/somtoolbox). A 5-by-5 neural network was initiated with hexagonal lattice structure. The input was the log-2 fold-change in protein synthesis following tunicamycin

treatment relative to DMSO controls for each time point. The network was randomly initiated and used Euclidean distance as the metric for classifying proteins to specific neurons. The SOM algorithm was repeated 1,000 times, and a co-clustering map was generated indicating the frequency with which any two proteins clustered in the same neuron. This co-clustering map was then subjected to hierarchical clustering using Euclidean distance as the metric for clustering proteins.

K-Means Clustering Analysis

K-means clustering was used to group proteins from EGF experiments into clusters with distinct temporal responses. Data was filtered by removing proteins that appeared in less than two out of four replicates, as well as removing proteins that did not show a statistically significant change (according to Student's t-test) in synthesis between EGF and PBS controls in at least one time point. After plotting the within-cluster distance against number of clusters, six clusters were selected for analysis, as increasing the number of clusters above six only marginally decreased the within-cluster distance. The input was log-2 fold-change in protein synthesis for all time points normalized to the 30 minute PBS control. Cluster centroids were initialized randomly, and Pearson correlation was used as the distance metric. K-means clustering was repeated 10,000 times, and a co-clustering map was generated indicating the frequency with which any two proteins shared the same cluster. This co-clustering map was then subjected to hierarchical clustering using Euclidean distance as the metric for clustering proteins.

Analysis of Temporal Delay Between RFP and MITNCAT Datasets

To prevent the analysis of random fluctuations in proteins with unchanging RFP values or protein synthesis rates, only proteins with at least one statistically significant time point (p<0.05) in both the RFP and MITNCAT datasets were considered, and thus only proteins present in at least two of the four MITNCAT replicates were included in the analysis of temporal delay. These

restrictions limited the protein data set to 90 and the RFP data set to 400; the overlap between these data sets was 27. Log2 PBS normalized values were centered around the mean and normalized to the standard deviation across time points. Because RFP analysis considers only 30, 60, and 90 minute time points whereas MITNCAT examines 30, 60, 90, 120, and 150 minute time points, MITNCAT values were standardized using the mean and standard deviation of the first three time points. Proteins were manually classified as "delayed" based on the relationship between the RFP and protein synthesis rate curves.

Codon Analytics

Human full-length open reading frames (ORFs) were obtained from the Mammalian Gene Collection (https://genecollections.nci.nih.gov/MGC/) [4]. Gene specific codon usage data was obtained for 32,751 human coding sequences using a described algorithm [5], which was previously used on yeast, rat and mouse genes and transcripts [6,7]. Briefly, human ORFS were computationally validated to ensure they contained start and stop codons and designated as gene sequences. Next all gene sequences were individually read from start to stop codon. The number of times each of the 64 possible in frame codons was used in each gene was recorded and used to determine gene specific codon frequencies, with the frequencies for all codons for a specific amino acid in a gene adding up to 1.00. Genome values for each codon frequency and standard deviation values were then obtained using data from the 32,751 analyzed genes. Codon usage frequencies from the "delayed" translation set were centered around the genome average and then averaged to calculate an average deviation for the set. The same calculation was performed for randomly generated sets when determining statistical significance (see *Statistical Methods)*.

To determine A-site enrichments for each codon, the codon in the A-site for each ribosome protected fragment (RPF) read was determined. After considering all read lengths from 24 to 40 nt, we found that the majority of our RPF reads were 30-35 nt long. Further, only reads between 30 and 35 nt long showed a strong enrichment for the triplet periodicity common in ribosome

profiling experiments. For these reasons, only reads of these lengths were considered for all RPF analyses. For these reads, the P site codon is at nucleotides 14, 15 and 16. The A-site is therefore at nucleotides 17, 18 and 19. We only considered reads where the A-site codon was in the reading frame of the coding sequence (approximately 60-65% of all reads). For each codon, the frequency with which it was in the A-site was recorded. The A-site frequency was then compared to the null expected frequency. This expected frequency was the frequency of each codon in the longest open reading frame of each gene weighted by the abundance of the gene in the ribosome profiling dataset. Enrichments were calculated as the observed frequency of a codon in A-sites compared to this expected background frequency. These enrichments were then calculated for every codon on 500 random subsets of 100 genes each. Based on their abundance in the 17 genes that displayed delayed increases in protein abundance (Figure 6B, upper right quadrant), codons were separated into "enriched" and "nonenriched" classes. The median A-site enrichment across codons in each class was calculated for each random subset.

Statistical Methods

For MITNCAT experiments, Student's t-test was used to calculate p-values comparing treated and control samples at all time points in GraphPad Prism. For RNA-Seq and RFP experiments, p-values were generated via the Wald Test using DESeq2. Translational Efficiency (TE) p-values were calculated using the Xtail algorithm [[3]]. Statistical significance was assigned for $p<0.05$. For codon bias analysis, empirical p-values were calculated using the random permutation test. Briefly, 1e6 random sets of genes with the same size as the query set were generated, and the codon frequency usage was calculated for that set. The p-value was calculated as the fraction of random sets with a more extreme codon frequency than the query set. Significance was assigned if p was less than the Bonferroni corrected α=7.81e-4 (corresponding to an expected FWER of 0.05 across the 64 unique codons). GO term enrichment was performed using the PANTHER classification system version 12.0 [[8]]. Cluster members were

queried against the background of all proteins included in the clustering analysis. P-values were generated from PANTHER.

Supplemental Methods References

1. Niers, J. M., Chen, J. W., Weissleder, R. & Tannous, B. A. Enhanced in vivo imaging of metabolically biotinylated cell surface reporters. *Anal. Chem.* **83,** 994–999 (2011).
2. Vizcaíno, J. A. *et al.* 2016 update of the PRIDE database and its related tools. *Nucleic Acids Res.* **44,** D447–D456 (2016).
3. Xiao, Z., Zou, Q., Liu, Y. & Yang, X. Genome-wide assessment of differential translations with ribosome profiling data. *Nat. Commun.* **7,** 11194 (2016).
4. Temple, G. F. The completion of the Mammalian Gene Collection (MGC). *Genome Res.* **19,** 2324–2333 (2009).
5. Begley, U. *et al.* Trm9-Catalyzed tRNA Modifications Link Translation to the DNA Damage Response. *Mol. Cell* **28,** 860–870 (2007).
6. Tumu, S., Patil, A., Towns, W., Dyavaiah, M. & Begley, T. J. The gene-specific codon counting database: A genome-based catalog of one-, two-, three-, four- and five-codon combinations present in Saccharomyces cerevisiae genes. *Database* **2012,** (2012).
7. Doyle, F. *et al.* Gene- and genome-based analysis of significant codon patterns in yeast, rat and mice genomes with the CUT Codon UTilization tool. *Methods* **107,** 98–109 (2016).
8. Mi, H. *et al.* PANTHER version 11: Expanded annotation data from Gene Ontology and Reactome pathways, and data analysis tool enhancements. *Nucleic Acids Res.* **45,** D183–D189 (2017).
